# Metal Manipulated Fluorescence: Mechanisms, Materials, and Plasmonic Strategies for Enhanced Emission

**DOI:** 10.3390/nano16050298

**Published:** 2026-02-26

**Authors:** G. Usha Nandhini, Manickam Minakshi, R. Sivasubramanian, Gnanaprakash Dharmalingam

**Affiliations:** 1 Plasmonic Nanomaterials Laboratory, Department of Nanoscience and Technology, PSG Institute of Advanced Studies, Coimbatore 641 004, Tamil Nadu, India; ug9707@srmist.edu.in; 2 College of Science, Health, Engineering and Education, Murdoch University, Perth, WA 6150, Australia; minakshi@murdoch.edu.au; 3 Department of Chemistry, Amrita School of Physical Sciences, Amrita Vishwa Vidyapeetham, Amaravati 522 240, Andhra Pradesh, India; s_subramanian@av.amrita.edu; 4 Department of Physics and Nanotechnology, SRM Institute of Science and Technology, Kattankulathur, Chennai 603 203, Tamil Nadu, India

**Keywords:** plasmon manipulated fluorescence, metal–fluorophore coupling, plasmon–fluorophore interactions

## Abstract

Fluorescence remains a foundational optical phenomenon underpinning applications in sensing, imaging, diagnostics, and catalysis. Among the strategies developed to modulate fluorescence, coupling fluorophores with plasmonic metals has emerged as a powerful route for both enhancement and quenching. The collective excitation and decay of surface plasmons can profoundly alter fluorophore excitation rates, radiative pathways, and emission efficiencies. This review provides a mechanistic and historical synthesis of metal–fluorophore interactions, unifying enhancement and quenching phenomena under the term Metal Manipulated Fluorescence (MMF). We summarize the fundamental principles of fluorescence and plasmon resonance, discuss theoretical and computational approaches for predicting metal–fluorophore coupling, and critically examine recent advances in plasmonic nanostructure synthesis that enable precise control over fluorophore behaviour. By integrating experimental observations with theoretical models, we highlight the opportunities and limitations of current MMF strategies and outline future directions in materials design, synthesis methodologies, and predictive modelling for next-generation optical and optoelectronic technologies.

## 1. Introduction

Metal-manipulated fluorescence (MMF), encompassing both surface-enhanced and plasmon-enhanced fluorescence, has evolved substantially since its first reports in the 1970s. With the rapid development of plasmonic nanostructures, MMF has become a versatile platform for sensing, imaging, and photonic applications. When fluorophores are positioned within the near field of a plasmonically active metal surface—typically between 5 and 90 nm—the local electromagnetic environment can significantly modify their excitation and emission behaviour. At very short distances (<5 nm), non-radiative energy transfer to the metal dominates, resulting in quenching. At intermediate distances, however, plasmon-mediated enhancement of excitation rates and radiative decay pathways can increase fluorescence intensities by several orders of magnitude. It is very interesting to note that even the gradients in electromagnetic fields in such sculpted structures can lead to interesting effects such as enhancements to intersystem crossing, crucial for fluorescence investigations [1].

Despite extensive experimental and theoretical work, the precise mechanisms governing fluorescence modification near metals remain debated due to the complexity of metal–fluorophore interactions. Surface plasmons, near-field electromagnetic enhancement, modified radiative rates, and energy-transfer pathways all contribute to the observed behaviour. To unify these diverse effects, we adopt the term Metal Manipulated Fluorescence (MMF), emphasizing that plasmonic structures can both enhance and suppress fluorescence depending on geometry, distance, and spectral overlap.

This review focuses on developments from the past decade, a period marked by significant advances in plasmonic nanostructure synthesis, enabling unprecedented control over morphology, composition, and optical response. Such progress has direct implications for MMF-based sensing, surface-enhanced Raman spectroscopy (SERS), and surface plasmon resonance (SPR) technologies [2]. A consolidated review of these developments is timely and essential for accelerating technological translation, guiding material design, and supporting interdisciplinary collaboration.

We structure this review as follows: Section 2 outlines the fundamental principles of fluorescence and its key quantification metrics. Section 3 discusses modelling approaches for predicting fluorescence behaviour and metal–fluorophore coupling. Section 4 provides a comprehensive overview of plasmon resonance physics, followed by Section 5, which connects plasmonic parameters to fluorescence manipulation. Section 6 surveys recent advances in MMF-active materials, organized by synthesis strategy, highlighting practical considerations such as cost, scalability, and structural tunability. Finally, we synthesize mechanistic insights from historical and contemporary studies and identify emerging opportunities in materials design and theoretical modelling.

## 2. Fluorescence and Its Governing Attributes

**Figure 1:**
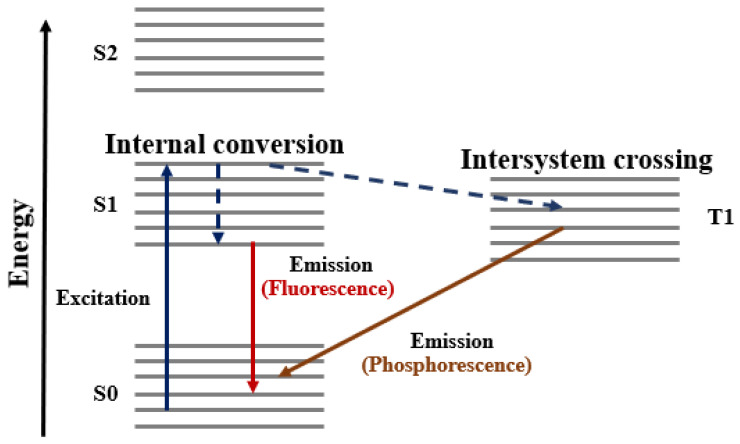
Jablonski diagram illustrating radiative and non-radiative pathways involved in fluorophore excitation and decay. The ground singlet electronic state is denoted as S_0_, with higher-energy excited singlet states represented by S_1_ and S_2_. The lowest-energy triplet state is T_1_. Vertical arrows indicate excitation and radiative decay processes, while curved arrows represent non-radiative relaxation pathways. The energy axis (E) reflects the relative ordering of electronic states.

George Gabriel Stokes first described the phenomenon of fluorescence in 1852 [3], observing that fluorite emits visible light when irradiated with ultraviolet radiation [4]. Fluorescence typically involves electronic transitions induced by light in the wavelength range of approximately 200–800 nm. It is fundamentally an electronic process in which certain molecules absorb photons of specific wavelengths characteristic of their electronic structure. Upon absorption, a subset of molecules is promoted from the ground electronic state (G) to an excited state (E), as illustrated in Figure 1. In the Jablonski diagram, horizontal lines represent electronic energy levels, while vertical or inclined arrows denote excitation and relaxation pathways. The upward arrow (A) corresponds to photon absorption and excitation, whereas the downward arrow (F) represents radiative relaxation, during which part of the absorbed energy is re-emitted as fluorescence.

The overall sequence, from photon absorption to emission, occurs on the order of 10^−8^ s. Although the electronic excitation itself is nearly instantaneous (∼10^−15^ s), the excited state may persist for several nanoseconds before relaxation. During this period, collisions and internal conversion processes dissipate part of the absorbed energy non-radiatively. Consequently, the emitted fluorescence photon has lower energy (longer wavelength) than the absorbed photon, a phenomenon known as the Stokes shift [5]. Fluorescence properties can be manipulated through various approaches. One effective approach is to improve the enhancement factor using a Purcell cavity [6]. The Purcell effect arises from an increase (or decrease) in the local density of photonic states at the emitter’s location, wherein the oscillator emits a wave that is reflected by the surrounding environment. The reflected wave then interacts with the oscillator: if it is in phase with the oscillator mode, radiation is suppressed; if it is out of phase, the damping rate increases, leading to enhanced radiation. Such nano and Pico cavities can also lead to significantly increased magnetic fields, leading to relaxation of the spin selection rules because of plasmon-induced spin–orbit coupling (PIST) [7]. In keeping in line with the context of this review, which is the explicit changes to fluorescence properties due to plasmon effects only, we have attempted to carefully ensure that the Purcell effect-mediated improvements in the enhancement factor and changes in fluorescent constants, such as lifetime, intensity, and yield, are not present in the reviewed literature. Indeed, separating the two enhancements is typically not strenuous as research generically investigates enhancements to fluorescence due to either of these two mechanisms and to the best of our knowledge, not both. Hence, this review focuses particularly on plasmon-mediated changes in fluorescence. A schematic representation of fluorescence processes is provided in Figure 1 for context.

The overall sequence, from photon absorption to emission, occurs on the order of 10^−8^ s. Although the electronic excitation itself is nearly instantaneous (∼10^−15^ s), the excited state may persist for several nanoseconds before relaxation. During this period, collisions and internal conversion processes dissipate part of the absorbed energy non-radiatively. Consequently, the emitted fluorescence photon has lower energy (longer wavelength) than the absorbed photon, a phenomenon known as the Stokes shift [8]. A schematic representation of these processes is provided in Figure 1 for context.

Several parameters are commonly used to quantify and evaluate fluorescence behaviour. In the following subsections, we describe the most relevant metrics: fluorescence lifetime (Section 2.1), molecular detection efficiency (Section 2.2), fluorescence quantum yield (Section 2.3), fluorescence enhancement factor (Section 2.4), and fluorescence intensity (Section 2.5). These parameters collectively provide a comprehensive framework for assessing the efficiency and dynamics of fluorophore emission.

### 2.1. Fluorescence Lifetime Analysis

Fluorescence lifetime measurements rely fundamentally on detecting and timing individual photons. Two principal approaches are widely used for this purpose: time-domain methods (Section 2.1.1) and frequency-domain methods (Section 2.1.2). Each provides complementary information about excited-state dynamics and is suited to different experimental contexts.

#### 2.1.1. Time-Correlated Single Photon Counting (TCSPC)

Time-correlated single photon counting (TCSPC) is one of the most precise and widely adopted techniques for determining fluorescence lifetimes. The method takes advantage of the fact that, under low-intensity, high-repetition-rate excitation, the probability of detecting more than one photon within a single excitation cycle is extremely small. Consequently, the system only needs to register the arrival time of individual photons relative to the excitation pulse rather than count multiple photons per cycle.

In TCSPC, each detected photon is assigned a time delay within the excitation period, and these delays are accumulated over many cycles to construct a histogram of photon arrival times. Most excitation cycles contain no detected photons, some contain exactly one, and only rarely does a cycle contain more than one photon.

Advanced single-photon detectors such as single-photon avalanche diodes (SPADs) and avalanche photodiodes (APDs) provide Instrument Response Function (IRF) widths in the range of 40–400 ps, with specialized APDs achieving values as low as 20 ps. Modern TCSPC systems further reduce the temporal channel width of the photon-arrival histogram to approximately 1 ps, enabling highly precise sampling of fluorescence decay profiles and facilitating the deconvolution of multi-exponential decay functions. Unlike time-gated detection methods, TCSPC records every detected photon with near-unity counting efficiency. The large number of temporal channels available in TCSPC minimizes statistical uncertainty in lifetime determination, yielding a near-optimal “Figure of Merit” for fluorescence decay measurements [9].

**Figure 2:**
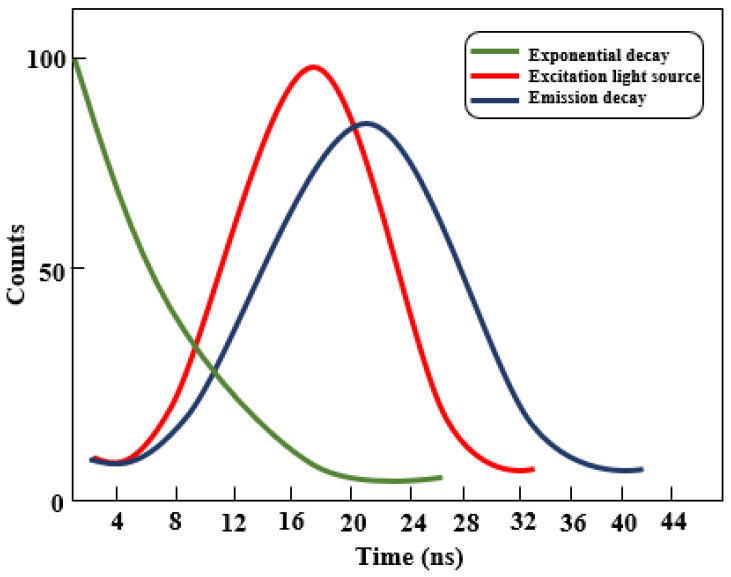
Fluorescence emission decay curves illustrating the TCSPC response of fluorophores under excitation. The exponential curve represents fluorescence lifetime, with the excitation and emission also shown for comparison.

Experimentally acquired decay curves are used to extract excited-state lifetimes. For fluorophores exhibiting single-exponential decay, the lifetime corresponds to the inverse of the slope of the logarithmic intensity–time plot (Figure 2). In systems containing multiple emissive species, the decay becomes multi-exponential and must be decomposed analytically into its constituent components. When the measured decay closely resembles the excitation pulse, distortion corrections are required. Lifetimes differing by ≤2 ns typically necessitate deconvolution to achieve adequate resolution.

To accomplish this, simulated composite decay curves—representing the convolution of the emission profile with the excitation source—are iteratively deconvoluted. The excitation pulse (lamp flash) is commonly modelled as a Gaussian function, while the emission decay is Gaussian prior to the peak and exponential thereafter. Deconvolution removes the contribution of the excitation pulse, isolating the intrinsic luminescence decay from which the single-exponential lifetime is obtained. The emission intensity is described by Equation (1):

(1) $$E\left(t\right)=E\left(0\right)e^{kt}$$

where *t* is the elapsed time, *k* is the decay constant, and *E*(*t*) and *E*(0) are the emission intensities at time t and time zero respectively. The lifetime is defined as the reciprocal of *k* [10].

#### 2.1.2. Frequency-Domain Methods for Lifetime Analysis

In frequency-domain fluorometry, the excitation light is sinusoidally modulated at high frequencies. Because the excited state persists for a finite duration, fluorophores subjected to such modulation emit fluorescence that is itself sinusoidally modulated but phase-shifted relative to the excitation source. This phase shift and the corresponding modulation depth form the basis for extracting fluorescence lifetime information. The emission intensity can be expressed as:

(2) $$I\left(t\right)=I_{0}[1+M_{F}\sin\left(\omega t+\Phi \right)]$$

where Φ is the phase delay between excitation and emission, ω is the angular modulation frequency, and I(t) and $I_{0}$ are the fluorescence intensities at time t and at t=0, respectively. One measure of the fluorescence lifetime, known as the phase lifetime $\tau_{p}$, is obtained from the phase delay using:

(3) $$\tan^{-1}\varphi =\omega \tau_{p}$$

The excitation (*M_E_*) and emission (*M_F_*) modulation depths are determined from the AC/DC ratios of the respective signals:

(4) $$M_{E}=\left(\frac{{AC}_{E}}{{DC}_{E}}\right)and\;M_{F}=\left(\frac{{AC}_{F}}{{DC}_{F}}\right)$$

The ratio of emission to excitation modulation is then:

(5) $$M=\frac{\left(M_{F}\right)}{\left(M_{E}\right)}$$

This ratio provides an alternative estimate of the fluorescence lifetime, known as the modulation lifetime *τ_m_*, through:

(6) $$M=\frac{1}{\sqrt{1+({\omega \tau }_{M}{)}^{2}}}$$

The waveforms may also be described using the sine (S) and cosine (G) components of the modulated emission:

(7) $$\phi =\tan^{-1}(S/G)$$

(8) M=(S2+G2)12

where

(9) $$S=\sum_{i}f_{i}M_{i}\sin\varphi_{i}$$

(10) $$M=\sum_{i}f_{i}M_{i}\cos\varphi_{i}$$

Here, $f_{i}$ and $M_{i}$ represent the fractional intensity and modulation depth of the $i^{\mathrm{th}}$ component respectively, with $\sum f_{i}=1$. Differences between $\tau_{M}$ and $\tau_{p}$ provide insight into lifetime heterogeneity, the presence of multiple emissive species, and the relative amplitudes of their decay components.

Practitioners of frequency-domain fluorometry typically describe how each component of the photocurrent contributes to the overall emission, whereas time-domain analyses refer to pre-exponential factors $\alpha_{i}$, which correspond to the relative populations of fluorescing species. Assuming that the quantum yields of the emitting species are proportional to their lifetimes, the relationship between $f_{i}$ and $\alpha_{i}$ becomes evident [11].

Instrumentally, time-correlated single photon counting (TCSPC) is often used to determine fluorescence lifetimes by fitting the intensity decay according to:

(11) $$I\left(t\right)=\sum_{i}^{k}\alpha_{i}e^{\frac{t}{\tau_{i}}}$$

where $\alpha_{i}$ are the pre-exponential factors, $\tau_{i}$ are the decay times, and k is the number of exponential components—commonly taken as three to balance accuracy and computational complexity. The fractional contribution of each component to the steady-state intensity is:

(12) $$f_{i}=\frac{\alpha_{i}\tau_{i}}{\sum_{i=1}^{k}\alpha_{i}\tau_{i}}$$

The mean (intensity-weighted) lifetime is:

(13) $$\bar{\tau }=\sum_{i=1}^{k}\alpha_{i}\tau_{i}$$

and the amplitude-weighted lifetime is:

(14) $$\;<\tau >\;=\;\sum_{i=1}^{k}\alpha_{i}\tau_{i}$$

Although both parameters are useful, the intensity-weighted lifetime is generally more reliable, as the amplitude-weighted lifetime is highly sensitive to fitting quality and model selection. A goodness-of-fit criterion ($\chi^{2}$) is typically used to refine $\alpha_{i}$ and $\tau_{i}$ through nonlinear least-squares reconvolution.

As an example, surface plasmon-coupled fluorescence (SPCF) studies have demonstrated significant lifetime modifications for fluorophores interacting with indium films coated with silicon dioxide [12]. Pyridine-2 exhibited lifetime reductions of 6.45% (free space) and 12.12% (glass). Tinopal-CBS showed decreases of 4.17% (glass) and 2.54% (free space), while sodium fluorescein displayed reductions of 21.51% (glass) and 15.44% (free space). These results highlight the strong influence of indium’s plasmonic properties on fluorescence lifetimes and underscore the potential of SPCF for enhancing analytical sensitivity [13].

### 2.2. Molecular Detection Efficiency

The relative molecular detection efficiency (MDE)—defined as the ratio of detected photons to absorbed photons—provides a useful measure of fluorescence detection performance. The MDE at a specific spatial position $r_{0}$, relevant for evaluating the detectability of fluorophores following excitation, is expressed as:

(15) $$MDE\;{(r}_{0})=\Gamma_{exc}\times Q\times MCE(r_{0})$$

Here, $\Gamma_{\mathrm{exc}}$ is the excitation rate of the fluorophore, Q is the fluorescence quantum yield, and MCE(r0) is the molecular collection efficiency at position $r_{0}$. Equation (15) highlights three primary strategies for improving fluorescence detection efficiency.

First, increasing the local excitation intensity enhances the electromagnetic field experienced by the fluorophore, thereby raising $\Gamma_{\mathrm{exc}}$. Second, modifying the internal relaxation pathways—such as by suppressing non-radiative decay—can increase the quantum yield Q. Third, improving the optical detection system enhances $\mathrm{MCE}(r_{0})$ by capturing a larger fraction of emitted photons.

Fluorophores can therefore be engineered or environmentally tuned to improve intensity and photostability through any combination of these three mechanisms. Nanomaterials, nanostructures, and nanostructured substrates play a particularly important role in this regard, as they can simultaneously enhance excitation fields, modify radiative rates, and improve photon collection [14].

For example, an optimized fluorescence collection system has been shown to increase collection efficiency by 50–90% compared with a non-optimized configuration at imaging depths of 1000 µm. This improvement enabled a 22% increase in excitation power, although simulations predicted enhancements approaching 100%. Such optimization facilitated clear in vivo imaging of layer V neurons at depths up to 850 µm [15].

### 2.3. Fluorescence Quantum Yield

Relative, absolute, and time-resolved methods for determining fluorescence quantum yield each offer distinct advantages and levels of accuracy depending on the experimental context. All approaches rely on specialized instrumentation—including calibrated light sources, detectors, and optical processing systems—to accurately capture and quantify emitted photons.

According to IUPAC, the integral quantum yield, Φ(λ), is defined as:

(16) $$\Phi \left(\lambda \right)=\frac{number\;of\;photons\;emitted}{number\;of\;photons\;absorbed}=\frac{N_{abs}}{N_{em}}$$

In addition to the fluorophore concentration and its molar (decadic) absorption coefficient $\varepsilon (\lambda_{\mathrm{ex}})$ at the excitation wavelength, the fluorescence quantum yield $\Phi_{f}$ characterizes the intrinsic photophysical properties of a chromophore.

Quantum yield can be determined using three principal methods:

(1) Relative measurement using the same excitation wavelength for both sample and standard (method 1a),
(2) Relative measurement using different excitation wavelengths (method 1b), and
(3) Absolute measurement, in which the number of emitted photons $N_{\mathrm{em}}$ is directly compared with the number of absorbed photons $N_{\mathrm{abs}}$, as in Equation (16).

Indirect approaches such as thermal lensing and photoacoustic spectroscopy may also be used, relying on quantification of dissipated heat.

Method 1a: Relative Quantum Yield Using the Same Excitation Wavelength

In method 1a, the sample’s quantum yield is determined relative to a standard of known quantum yield, with all experimental conditions—temperature, solvent, and excitation wavelength—carefully matched. The relative quantum yield is calculated using the Demas and Crosby relation:

(17) $$\Phi_{f,x}=\;\Phi_{f,st\;}\frac{F_{x}}{F_{st}}\frac{f_{st\;}(\lambda_{ex,st})}{f_{x}(\lambda_{ex,x})}\frac{n_{x}^{2}}{n_{st}^{2}}\frac{q_{p,st\;}(\lambda_{ex,st})}{q_{p,x}\;(\lambda_{ex,x})}$$

where subscripts *x* and *st* denote sample and standard, respectively; *ex* and *em* refer to excitation and emission wavelengths; and $\Phi_{f,st}$ is the standard’s known quantum yield.

The absorption factor $f(\lambda_{\mathrm{ex}})$, representing the fraction of excitation light absorbed, is given by:

(18) $$f{(\lambda }_{ex})=1-10^{-A(\lambda_{ex})}$$

Photon fluxes $q_{p,st}$ and $q_{p,x}$ must be considered when different excitation wavelengths are used (method 1b), but are identical in method 1a.

Method 1b: Relative Quantum Yield Using Different Excitation Wavelengths

When the sample and standard require different excitation wavelengths, refractive index corrections and wavelength-dependent photon flux adjustments must be applied. The quantum yield becomes:

(19) $$\Phi_{f,x}=\Phi_{f,st}\prod_{i=1}^{n}\frac{F_{x,i}}{F_{st,i}}\frac{f_{st,i}}{f_{x,i}}\frac{\left(\lambda_{ex,st}\right)}{\left(\lambda_{ex,x}\right)}\frac{n_{x,i}^{2}(\lambda_{em,x})}{n_{st,i}^{2}(\lambda_{em,st})}=\;\Phi_{f,st\;}\prod_{i=1}^{n}C_{i}$$

This formulation accounts for differences in excitation wavelength, solvent refractive index, and spectral response.

Method 2: Absolute Quantum Yield

Absolute quantum yield measurements require only that the sample and standard be measured under identical instrument settings. Both emission and excitation corrections must be applied. Time-resolved quantum yield determination further improves accuracy by incorporating fluorescence lifetime information, thereby capturing dynamic emission behaviour.

Quantum Yield in the Context of Plasmon-Enhanced Fluorescence

To connect these measurement approaches with plasmon-enhanced fluorescence, it is essential to consider how the quantum yield changes in the presence of plasmonic near-fields. A widely cited relationship suggests that fluorescence enhancement in the near field is inversely proportional to the free-space quantum yield, i.e., MMF ≈ 1/$Q_{0}$.

In a homogeneous medium, the intrinsic quantum yield is:

(20) $$Q_{0}=\frac{\Gamma }{\Gamma +K_{nr}}$$

where Γ is the radiative decay rate and $K_{nr}$ is the non-radiative decay rate. The corresponding free-space lifetime is:

(21) $$\tau_{0\;}=\frac{1}{\Gamma +K_{nr}}$$

In the near field of a metallic nanoparticle, an additional radiative decay channel $\Gamma_{m}$ is introduced, modifying the quantum yield to:

(22) $$Q_{m}=\frac{\Gamma +\Gamma_{m}}{\Gamma +\Gamma_{m}+K_{nr}}$$

The associated lifetime becomes:

(23) $$Q_{m}=\frac{1}{\Gamma +\Gamma_{m}+K_{nr}}$$

Equations (22) and (23) show that increasing $\Gamma_{m}$ simultaneously increases the quantum yield and decreases the lifetime, consistent with classical far-field behaviour (Equations (20) and (21)). These relationships support experimental observations that metal-manipulated fluorescence enhancement factors are inversely proportional to $Q_{0}$. Consequently, they are essential for estimating optimal fluorophore–metal distances for controlled emission modification [16,17,18].

For example, reported quantum yield ratios (RΦ) include:

- Rose bengal: 0.10–0.15 (10–15%)
- Rhodamine B: ~95%
- Dipyridamole: ~5% at pH 3 and ~80% at pH 8 [19]

These values illustrate the wide variability in fluorophore quantum yields and the importance of environmental conditions.

### 2.4. Fluorescence Enhancement Factor (EF)

In MMF systems, the fluorescence enhancement factor (EF) is commonly defined as the ratio of the modified quantum yield $Q_{m}$ (Equation (23)) to the intrinsic quantum yield $Q_{0}$ (Equation (20)). This ratio quantifies the degree to which a plasmonic nanostructure improves the fluorescence efficiency of a nearby fluorophore. Mathematically, EF is expressed as:

(24) $$EF=\;\frac{Q_{m}}{Q_{0}}=\frac{\frac{1}{\Gamma +\Gamma_{m}+K_{nr}}}{\frac{\Gamma }{\Gamma +K_{nr}}}=\;\frac{\left(\Gamma +\Gamma_{m})(\Gamma +K_{nr}\right)}{\Gamma (\Gamma +\Gamma_{m}+K_{nr})}$$

This enhancement factor is central to determining optimal fluorophore–metal distances for maximizing fluorescence enhancement and provides insight into how plasmonic fields modify radiative and non-radiative decay pathways.

The fluorescence enhancement is closely linked to the optical response of the plasmonic nanoparticle, particularly its absorption ($C_{a}$) and scattering ($C_{s}$) contributions to the total extinction cross-section $C_{E}$, given by:

(25) CE= CA+Cs= K1Im (α) + K146π|α2|

where $K_{1}=\frac{2\pi n_{1}}{\lambda_{0}}$ is the wave vector of the incident light, α is the nanoparticle polarizability, r is the particle radius, $n_{1}$ is the refractive index of the surrounding medium, and $\lambda_{0}$ is the incident wavelength. The polarizability of a spherical nanoparticle is:

(26) $$\alpha \;=\;4\pi r^{3}(\varepsilon_{m}-\;{\;\varepsilon }_{1})/(\varepsilon_{m}+\;2\varepsilon_{1})$$

where $\varepsilon_{1}$ is the dielectric constant of the medium and $\varepsilon_{m}$ is the complex dielectric function of the metal.

Because the scattering term scales with $r^{6}$ and the absorption term with $r^{3}$, larger nanoparticles generate significantly stronger scattering-dominated near fields. Consequently, fluorophores positioned near larger plasmonic nanoparticles typically experience greater enhancement than those near smaller particles [20].

Experimentally, enhancement factors of 2–4 have been reported for fluorophores such as fluorescein and IR-780 when coupled to Ag and Au nanoparticles, with enhancement saturating at excitation powers around 50 mW [21]. These observations underscore the importance of nanoparticle size, dielectric environment, and excitation conditions in determining MMF performance.

### 2.5. Fluorescence Intensity

**Figure 3:**
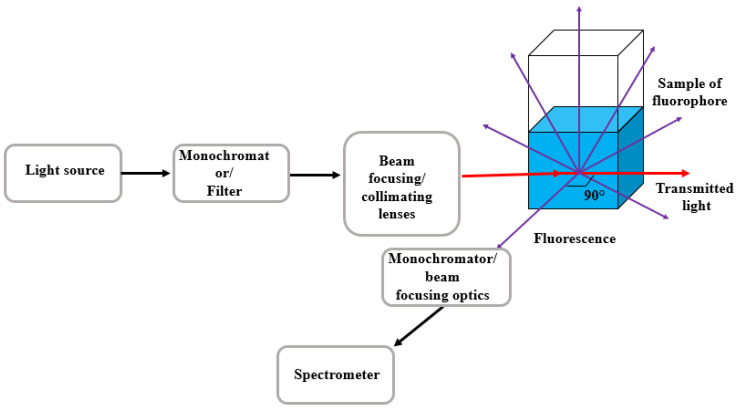
Schematic of a generic fluorescence acquisition setup. The system typically includes an excitation light source, optical filters, a sample holder, and a detection pathway incorporating lenses, mirrors, and a photodetector. Together, these components enable controlled excitation of the fluorophore and efficient collection of the resulting emission signal.

In a typical fluorescence measurement setup, the emission collection optics are positioned perpendicular to the excitation beam to minimize interference from scattered excitation light. The emitted fluorescence is spectrally selected using a grating-based monochromator, which isolates the desired emission wavelength range. The essential components of a standard fluorescence acquisition system are illustrated in Figure 3; these elements form the core of the detection pathway, although additional optical components may be incorporated to enhance signal collection, sensitivity, or spectral resolution.

Two general approaches are commonly used to measure fluorescence intensity. In the first, the excitation wavelength is held constant while the emission spectrum is scanned over a selected wavelength range. In the second, the emission wavelength is fixed and the excitation wavelength is scanned. According to the Beer–Lambert law, fluorescence intensity is proportional to the concentration of the fluorophore. However, because only a fraction of absorbed photons contribute to fluorescence, the measured intensity is proportional to the number of absorbed photons multiplied by the fluorophore’s quantum yield.

Direct comparison of fluorescence intensities between different samples is complicated by fluctuations in excitation photon flux and by variations in the fraction of emitted photons collected by the detection system. Therefore, appropriate corrections must be applied—specifically, normalization to the excitation intensity and correction for the collection efficiency of the optical system. These considerations lead to the expression given in Equation (27).

(27) $$F{(\lambda }_{em})=P_{0}{(\lambda }_{exc})\varepsilon {(\lambda }_{exc})bc\Phi K{(\lambda }_{exc},\lambda_{em})$$

where the observed fluorescence intensity F, depends on several parameters, including the molar absorption coefficient (ε), optical path length (b), fluorophore concentration (c), fluorescence quantum yield (Φ), excitation light intensity $\left(P_{0}\right)$, and an instrument-specific calibration factor (K). Most of these parameters exhibit wavelength dependence with respect to both excitation and emission wavelengths $\left(\lambda_{ex},\lambda_{em}\right)$ [22,23,24]. As an example, placing perylene in close proximity to silver island films (SiFs) increased its fluorescence intensity by nearly 90%, raising its glass-normalized value from 1.0 to approximately 2.0 due to Ag nanoparticle–surface plasmon interactions [25]. Such observations highlight the importance of these parameters as benchmarks for evaluating the optical efficiency of fluorophores.

The parameters described thus far represent the most commonly quantified metrics in fluorescence research. To accelerate the identification of optimal fluorophore–nanostructure configurations, predictive modelling becomes highly advantageous. Computational tools play a critical role in this context, enabling both qualitative and quantitative simulation of the optical behaviour of materials and structures prior to experimental realization. As a result, reliance on computational approaches has become essential, and careful consideration of the capabilities, limitations, and suitability of different modelling tools is necessary. In the following section, we discuss the most widely used simulation techniques, their applicability, expected outputs, and the metrics by which they are evaluated.

## 3. Modeling of Fluorescent Behavior

Theoretical modelling plays a crucial role in quantifying and predicting fluorescence behaviour, particularly when multi-exponential decay dynamics are involved. By fitting fluorescence lifetime components, tools such as Decay-Associated Spectroscopy (DAS) and software platforms like TemPro enable detailed analysis of complex decay profiles and facilitate the extraction of meaningful photophysical parameters.

Because surface plasmon resonance can significantly modify fluorescence intensity, lifetime, and quantum yield through both near-field and far-field interactions, it is highly advantageous to visualize these effects prior to material fabrication. Computational modelling provides this capability. Techniques such as finite-difference time-domain (FDTD) simulations allow researchers to predict spectral overlap conditions, fluorophore–metal distance dependencies, and the resulting enhancement or quenching behaviour induced by plasmonic nanostructures.

By correlating these simulations with experimental observations—typically obtained through time-resolved fluorescence spectroscopy—researchers can establish a direct link between predicted electromagnetic field distributions and measured luminescence responses. Such modelling therefore serves as a powerful tool for guiding the design, optimization, and interpretation of metal–fluorophore systems.

### 3.1. Multi-Exponential Modeling Using DAS Software

Powerful modelling tools such as Decay-Associated Spectroscopy (DAS) software enable researchers to analyse complex luminescence phenomena and extract meaningful insights into material properties, biomolecular interactions, and chemical reaction mechanisms. In the context of fluorescence, DAS is particularly valuable for deconvoluting spectral data in which intensities and lifetimes are altered due to changes in radiative and non-radiative decay rates. This is accomplished by resolving the fluorescence decay into multiple lifetime components, typically denoted as α_1_, α_2_, and α_3_.

These components form the basis of the multi-exponential decay model used to describe Metal-Manipulated Fluorescence (MMF). The short-lived component (α_1_), typically in the range of ~0.1–1 ns, is associated with non-radiative decay pathways, quenching processes, and metal-induced relaxation. The medium-lived component (α_2_), generally ~10–100 ns, corresponds to radiative decay, fluorescence emission, and metal-enhanced emission. The long-lived component (α_3_), spanning ~1–100 µs, is linked to metal-induced triplet-state relaxation, phosphorescence, and triplet-state formation. Together, these components provide detailed information about fluorophore–metal interactions and allow researchers to tailor MMF behaviour for specific applications.

The average decay lifetime is expressed as the intensity-weighted mean fluorescence lifetime (Equation (13)), calculated using the pre-exponential factors αᵢ. The amplitude-weighted lifetime (Equation (14)), which places greater emphasis on longer-lived components, often serves as a more representative measure of fluorescence activity. The fractional contribution of each decay component is given by Equation (12). Collectively, these multi-exponential lifetime parameters are essential for analysing and interpreting fluorophore–metal coupling.

According to the multi-exponential decay model, the fluorescence intensity as a function of time is:

(28) $$I\left(t\right)=\alpha_{1}\exp(t/\tau_{1})+\alpha_{2}\exp(t/\tau_{2})+\alpha_{3}\exp(t/\tau_{3})\;\;\;$$

where I(t) is the fluorescence intensity at time t, α_1_, α_2_, and α_3_ are the amplitudes of the short-, medium-, and long-lived components, and τ_1_, τ_2_, and τ_3_ are their corresponding lifetimes. While the mean and amplitude-weighted lifetimes provide simplified descriptors of decay behaviour, Equation (28) captures the full temporal evolution of the fluorescence signal. Together, these analytical tools enable comprehensive interpretation of fluorophore–metal interactions and are widely used in MMF research [26,27].

### 3.2. Tempro Fluorescence Software

Another modelling tool that provides a comprehensive perspective on fluorescence behaviour is the TemPro fluorescence lifetime system. TemPro can be used to measure fluorescence intensity decay functions for samples deposited on different substrates. Its operation typically involves sample preparation, pulsed-laser excitation, and fluorescence detection using photomultiplier tubes (PMTs) or avalanche photodiodes (APDs). In this system, phase-modulation techniques quantify phase shifts in the emission, while TCSPC records individual photon arrival times.

Using DAS software, the excited-state lifetimes ($\tau_{i}$) and their corresponding amplitudes ($\alpha_{i}$) can be extracted from multi-exponential decay fits. TemPro then computes the amplitude-weighted fluorescence lifetime, which accounts for the contributions of multiple decay pathways, each represented by an exponential component. This provides a concise yet informative descriptor of the system’s fluorescence behaviour:

(29) $$<\tau >\;=\;\sum_{i=1}^{n}\alpha_{i}\times \tau_{i}$$

where n is the number of decay components in the fluorescence decay function.

Although Equation (29) and the multi-exponential decay expression (Equation (46)) serve different purposes, both describe fluorescence decay. The multi-exponential model captures the full time-dependent decay behaviour, reflecting how fluorescence intensity decreases due to multiple radiative and non-radiative processes. In contrast, the amplitude-weighted lifetime condenses this behaviour into a single representative value, simplifying interpretation while still reflecting the underlying decay dynamics. Both quantities are derived from the same pre-exponential factors and decay times: Equation (13) provides the mean lifetime, and Equation (12) gives the fractional contribution of each decay component.

While both TemPro and DAS analyse fluorescence lifetimes, their scopes differ. DAS software is typically used in material and biomolecular studies to extract mean and amplitude-weighted lifetimes from multi-exponential decay curves obtained via TCSPC. TemPro, on the other hand, is optimized for real-time fluorescence lifetime measurements and is often preferred for instrument-specific or rapid-acquisition applications. In essence, TemPro provides streamlined, hardware-integrated lifetime analysis, whereas DAS offers more comprehensive analytical capabilities, even though both rely on TCSPC principles [27,28].

With this overview of computational tools for modelling pristine and metal-modified fluorescence behaviour, we now turn to the central theme of this review: plasmonic materials and their ability to manipulate fluorescence. The following section focuses specifically on aspects of plasmon resonance that directly influence fluorescence enhancement and quenching, rather than providing an exhaustive treatment of plasmonic physics.

## 4. Surface Plasmon Resonance

Surface plasmon resonance (SPR) arises when polarized light interacts with a metal surface at a specific angle, exciting collective oscillations of free electrons at the metal–dielectric interface. These oscillations give rise to characteristic electromagnetic modes that strongly influence light–matter interactions at sub-wavelength scales. In the following subsections, we outline the governing principles of SPR, beginning with the theory of plasmons (Section 4.1), followed by propagating surface plasmon polaritons (Section 4.2), and localized or standing-wave plasmons (Section 4.3).

### 4.1. Theory of Plasmons

Although the interaction of light with free-carrier-rich materials has been observed for centuries, the field describing these interactions has only recently been formalized under the term plasmonics [29]. Under appropriate conditions, incident electromagnetic radiation can excite surface plasmons (SPs)—collective oscillations of conduction electrons at a metal–dielectric interface. These resonant oscillations generate tightly confined electromagnetic waves. When these waves propagate along the interface, they are referred to as surface plasmon polaritons (SPPs). When the oscillations are confined to discrete metallic nanostructures, they are known as localized surface plasmons (LSPs) [30].

A related excitation, the bulk plasmon, involves longitudinal charge oscillations within the volume of a material. However, because bulk plasmons require longitudinal electric fields, they cannot be excited by light (which carries only transverse fields) and instead require electron bombardment. As bulk plasmons fall outside the scope of this review, readers are referred to detailed treatments in the literature [30,31].

The resonance characteristics of plasmons are highly sensitive to changes in material shape, composition, and local dielectric environment [32,33,34,35]. The bulk plasmon resonance frequency, $\omega_{p}$, is given by:

(30) $$\omega_{p}^{2}=\frac{n_{e}^{2}}{\varepsilon_{0\;}m}$$

A detailed derivation of $\omega_{p}$ can be found in standard plasmonics references.

Once excited, plasmon propagation and damping are governed by the material’s complex relative permittivity. For metals—ideal candidates for plasmonic excitation due to their high free-electron densities—the Drude model provides a good approximation of the dielectric function:

(31a) $$\varepsilon_{m}=1-\frac{\omega_{p}^{2}}{\omega^{2}-i\gamma \omega }$$

where γ is the relaxation frequency, representing the inverse of the average time between electron scattering events. Coinage metals such as Ag, Au, and Cu are widely used in plasmonics due to their favourable optical properties. However, many other materials—including doped semiconductors, nitrides, and even carbon—can exhibit plasmonic behaviour when engineered to possess sufficiently high charge-carrier densities, enabling plasmon resonances extending into the ultraviolet [30,36,37].

**Figure 4:**
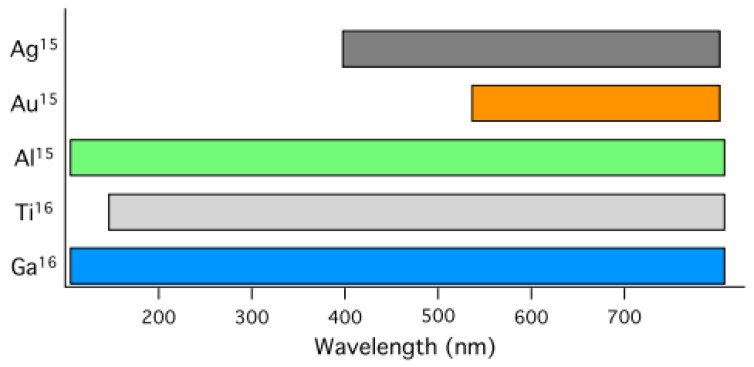
Graphical representation of the surface plasmon resonance tuning ranges of common plasmonic metals. The diagram highlights the spectral regions accessible through materials such as Ag, Au, Ti, Ga, and Al, illustrating how their intrinsic dielectric properties determine the wavelengths at which strong plasmonic responses occur. Consequently, MMF enhancement can be achieved. Reproduced with permission from “Recent advances in plasmonic nanostructures for sensing: a review, Pietro Strobbia, Eric R. Languirand, Brian M. Cullum, Optical Engineering, Vol. 54, Issue 10, 100902 (October 2015). https://doi.org/10.1117/1.OE.54.10.100902.” [30].

Different plasmonic materials support SP resonances across distinct spectral ranges. As illustrated in Figure 4, aluminium supports plasmons across a broad portion of the electromagnetic spectrum, albeit with lower resonance intensities compared to Ag and Au. In contrast, Au and Ag exhibit strong, well-defined plasmon resonances primarily in the visible and near-infrared (NIR) regions [30].

The optimal energy required to excite a surface plasmon is strongly influenced not only by the composition of the plasmonic material but also by its geometry, spatial distribution, and the dielectric properties of its surrounding environment [30]. These factors collectively determine the resonance conditions and the spectral position of the plasmonic response.

Building on this primer on surface plasmons, we now provide a concise yet technically detailed overview of the two principal classes of surface plasmons: propagating surface plasmon polaritons (SPPs) and localized surface plasmons (LSPs).

### 4.2. Surface Plasmon Polaritons (SPPs)

Surface plasmon polaritons (SPPs) have a rich historical foundation. Their earliest observation dates back to 1902, when Wood reported unusual optical reflection features from metallic gratings. In 1908, Mie provided the theoretical solution for light scattering by spherical particles, explaining the vivid colours observed in metal-doped glasses by Maxwell Garnett and others. In 1956, Pines proposed a theoretical framework describing the characteristic energy losses experienced by fast electrons traversing metals, identifying these losses as “plasmons”—collective oscillations of free electrons—and unifying concepts of surface and bulk plasmons. In the same year, Fano introduced the term “polariton” to describe the coupled oscillation of light and bound electrons in transparent materials.

Subsequent work by Ritchie revealed that thin metallic films exhibit anomalous electron-energy-loss features arising from surface plasmon excitation, confirming that plasmon modes could exist at metal surfaces [38]. Otto and Kretschmann later demonstrated optical excitation of surface plasmons on metal foils, establishing the foundations of prism-coupling techniques. The surface plasmon properties of Au and Ag nanoparticles were systematically characterized by Kreibig and Zacharias, and shortly thereafter, Cunningham and co-workers introduced the term surface plasmon polariton (SPP), now widely used to describe these hybrid light–electron surface waves.

SPP-based structures and devices exhibit remarkable properties relevant to solar energy harvesting, optical data storage, integrated photonics, surface-enhanced Raman spectroscopy (SERS), and chemical and biological sensing. Notably, SPPs can generate near-field enhancements 100–200 times stronger than the incident electromagnetic field, and SPP-active Ag nanoparticles have demonstrated sensitivities up to 60 times higher than conventional fluorescent labels [39,40].

**Figure 5:**
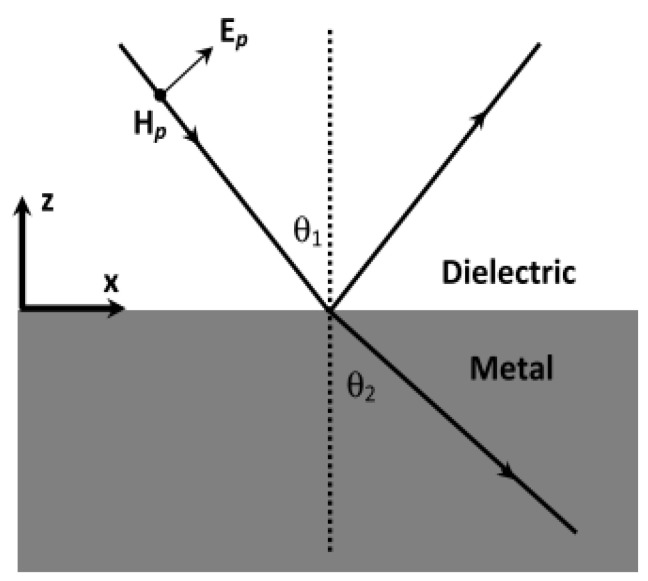
Representation of p-polarized electromagnetic radiation incident at an angle of incidence $\theta_{1}$ onto a planar interface between two media. The diagram illustrates the orientation of the electric field vector relative to the plane of incidence and highlights the boundary conditions governing reflection and transmission at the interface. Reproduced with permission from “Surface plasmon polaritons: physics and applications, Junxi Zhang, Lide Zhang and Wei Xu, J. Phys. D: Appl. Phys. 45, 113001, DOI 10.1088/0022-3727/45/11/113001.” [40].

To formulate the characteristics of SPPs, consider a p-polarized (transverse magnetic, TM) electromagnetic wave incident on a smooth planar metal–dielectric interface at an angle $\theta_{1}$, as illustrated in Figure 5. The incident photon carries momentum $\hbar k_{d}$, where:

(31b) $$k_{d}=\frac{2\pi n_{d}}{\lambda }$$

and $n_{d}$ is the refractive index of the dielectric. The reflected wave propagates at the same angle $\theta_{1}$, while the refracted wave inside the metal propagates at an angle $\theta_{2}$. Under appropriate conditions, the in-plane component of the incident wavevector matches that of the surface plasmon mode, enabling excitation of an SPP at the interface.

Since the refractive index of the metal is denoted by $n_{m}$, the photon momentum within the metal is $\hbar k_{m}$. Conservation of the in-plane (x-direction) momentum component requires that:

(32) $$n_{d}\;sin\theta_{1}=n_{m}\;sin\theta_{2}$$

The dispersion relation for a surface plasmon polariton (SPP) at a metal–dielectric interface is given by:

(33) $$k_{spp}=k\sqrt{\frac{\varepsilon_{d}\varepsilon_{m}}{\varepsilon_{d}+\varepsilon_{m}}}$$

**Figure 6:**
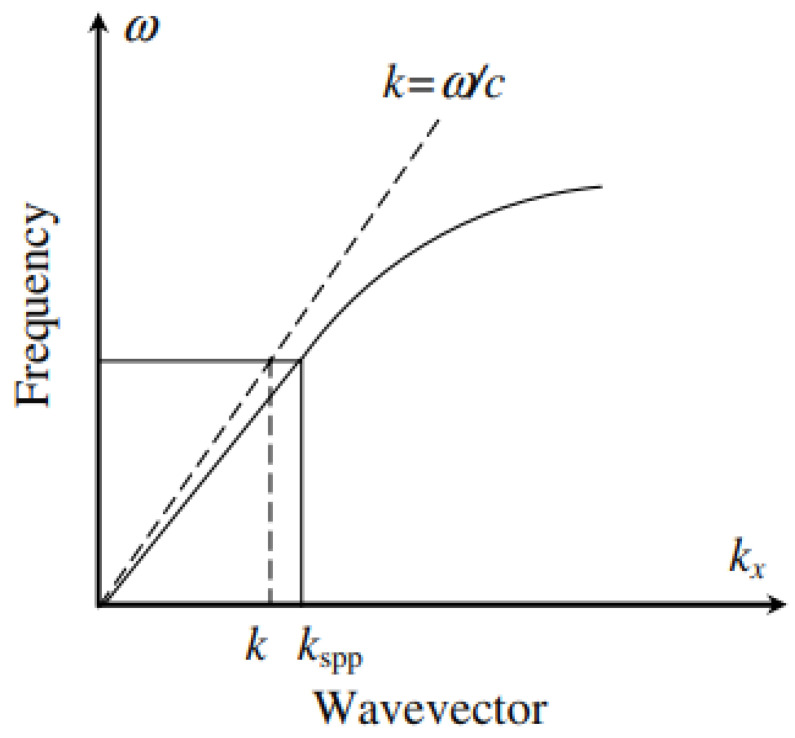
Comparison of the Momentum $\left(\hbar k_{spp}\right)$ of a surface plasmon polariton (SPP) wave with that (ℏk) of a free-space photon at the same frequency (ω). Here, k_spp_ and k denote the SPP and free-space wave vectors, respectively, illustrating that SPPs possess larger in-plane momentum than photons of identical frequency. Reproduced with permission from “Surface plasmon polaritons: physics and applications, Junxi Zhang, Lide Zhang and Wei Xu, J. Phys. D: Appl. Phys. 45, 113001, DOI 10.1088/0022-3727/45/11/113001.” [40].

As illustrated in Figure 6, the SPP dispersion curve exhibits a nonlinear behaviour, resulting in a momentum mismatch between incident photons and the SPP mode. Specifically, the SPP momentum $\hbar k_{\mathrm{spp}}$ exceeds that of a free-space photon ℏk at the same frequency. Consequently, direct excitation of SPPs by incident light is not possible without additional momentum-matching mechanisms. This mismatch is typically overcome using coupling strategies such as diffraction gratings, prism-based configurations, or engineered surface roughness, all of which provide the additional in-plane momentum required to excite SPPs at the interface.

The penetration depths of the SPP electric field into the dielectric and metal, denoted $\delta_{d}$ and $\delta_{m}$, are defined as $\delta_{d}=1/k_{zd}$ and $\delta_{m}=1/k_{zm}$, respectively, corresponding to the distances over which the fields decay to 1/e of their interface value. The penetration depth of the SPP field into the dielectric is:

(34) $$\delta_{d}=\frac{1}{k}{\left|\frac{\varepsilon_{d}+\varepsilon_{m}}{{-\varepsilon }_{d}^{2}}\right|}^{\frac{1}{2}}$$

While the penetration depth into the metal is

(35) $$\delta_{m}=\frac{1}{k}{\left|\frac{\varepsilon_{d}+\varepsilon_{m}}{{-\varepsilon }_{m}^{2}}\right|}^{\frac{1}{2}}$$

**Figure 7:**
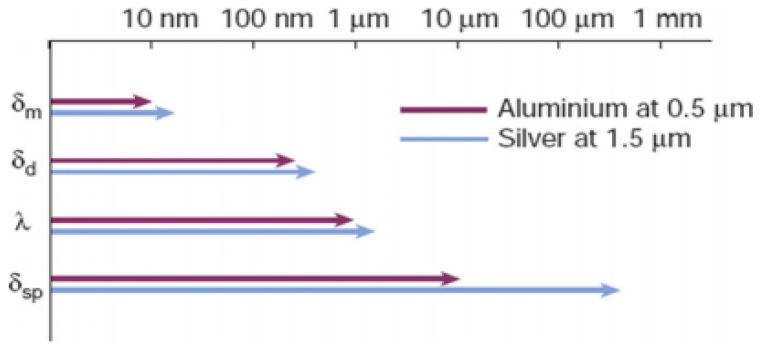
Comparison of surface plasmon polariton (SPP) propagation lengths in aluminium (Al) and silver (Ag). The plot highlights the significantly longer propagation distances supported by Ag across much of the visible and near-infrared spectrum, reflecting its lower intrinsic losses relative to Al. Reproduced with permission from “Surface plasmon polaritons: physics and applications, Junxi Zhang, Lide Zhang and Wei Xu, J. Phys. D: Appl. Phys. 45, 113001, DOI 10.1088/0022-3727/45/11/113001.” [40].

Because both penetration depths are relatively small, the SPP electric fields are strongly confined to the vicinity of the metal–dielectric interface. This intense field localization is highly advantageous for sensitively probing surface-adsorbate interactions. The dielectric properties of the surrounding medium play a dominant role in determining SPP propagation behaviour, leading to significant variations in propagation length, as illustrated in Figure 7.

Beyond single-interface SPPs, multilayer thin-film structures can be engineered to support coupled SPP modes at multiple interfaces. Such coupling gives rise to symmetric and antisymmetric SPP modes. The symmetric mode—commonly referred to as the long-range SPP (LRSPP)—can exhibit propagation lengths dramatically longer than those of single-interface SPPs, in some cases by factors approaching 138. Extensive literature is available for readers interested in the design and exploitation of multilayer films for exciting these advanced plasmonic modes [40].

With this brief overview of SPPs, we now turn to localized surface plasmons (LSPs). As will be shown, LSPs can be equally—if not more—significant than their propagating counterparts in the context of MMF studies, offering powerful advantages for both fundamental investigations and practical applications.

### 4.3. Localized Surface Plasmon Resonance (LSPR)

Localized surface plasmon resonance (LSPR) is a nanoscale optical phenomenon that has been extensively studied due to its remarkable ability to generate strong electromagnetic near-field enhancements and pronounced spectral absorption and scattering. These resonances arise when the conduction electrons in a metallic nanoparticle oscillate collectively in response to incident light. The analytical description of LSPR—its physical origin and its dependence on key parameters such as particle size, shape, composition, and the refractive index of the surrounding medium—is outlined below.

A natural starting point for deriving the LSPR condition is the relationship between the displacement D of a free-electron gas and the incident electric field E that drives it. This classical electron-oscillator model forms the basis for obtaining the LSPR frequency of a free-electron cloud, expressed as:

(36) $$D=\varepsilon_{0}E+P$$

The polarization density is denoted by P. Solving the classical equation of motion for an individual free electron subjected to an oscillating electric field yields:

(37) $$P=\frac{{-ne}^{2}}{m\left(\omega^{2}+i\gamma \omega \right)E}$$

where m is the effective electron mass, ω is the angular frequency of the applied electric field, and γ represents damping or dissipation. The corresponding relation between the dielectric displacement D and the external electric field is:

(38) $$D=\varepsilon_{0}\left(1-\frac{\omega_{p}^{2}}{\omega^{2}+i\gamma \omega }\right)E$$

where the plasma frequency of the electron cloud is $\omega_{p}=\sqrt{\frac{e^{2}n}{\varepsilon_{0}m}}$.

Equation (38) represents the general constitutive relation for a linear isotropic material. Near the plasma frequency, damping becomes negligible (γ≈0), allowing the LSPR frequency to be expressed as [36,41,42]:

(39) $$\omega_{max}=\;\frac{\omega_{p}}{\sqrt{{2e}_{m\;}+1}}\;$$

Thus, the LSPR frequency is strongly dependent on the free-electron density and the dielectric constant of the surrounding medium for a given plasmonic nanoparticle.

Under the quasistatic approximation, the absorption cross-section of a spherical plasmonic nanoparticle is given b:

(40) $$C_{a}=\frac{{4\pi }^{2\;}R^{3}{N\epsilon }_{s}^{3/2}}{\lambda }\frac{\varepsilon_{i}}{(\varepsilon_{r+}{2\varepsilon }_{s}{)}^{2}+\varepsilon_{2}^{2}}$$

where N is the conduction electron density, R is the particle radius, $\varepsilon_{s}$ is the dielectric function of the surrounding medium, and $\varepsilon_{r}$ and $\varepsilon_{i}$ are the real and imaginary parts of the nanoparticle’s dielectric function.

At resonance, absorption is maximised when:

(41) $$\varepsilon_{r}=-{2\varepsilon }_{s}$$

Substituting this condition into Equation (39) yields:

(42) $$\omega =\frac{\omega_{p}}{\sqrt{{2\varepsilon }_{s}+1}}$$

It is important to note that the factor of 2 in Equation (41) applies only to spherical nanoparticles. For anisotropic morphologies such as nanorods, this factor is replaced by $(1-p_{j})/p_{j}$, where $p_{j}$ is the depolarization factor determined by the aspect ratio. Using Gans’ theory—which treats nanorods as prolate spheroids with three principal axes—the extinction coefficient for randomly oriented nanorods can then be derived accordingly.

(43) $$\gamma =\frac{2NV{\pi \varepsilon }_{m}^{3/2}}{3\lambda }\sum_{j}\frac{(1/P_{j}^{2})\varepsilon_{2}}{(\varepsilon_{1}+\left(1-P_{j}\right)\frac{1}{P_{j}}\varepsilon_{m}{)}^{2}+\varepsilon_{2}^{2}}$$

where V is the particle volume and $P_{j}$ the depolarization factor is given by,

(44) $$P_{A}=\left[\left(1-e^{2}\right)/e^{2}\right]\left[1/2e\;In\;(\left(1+e\right)/(1-e))-1\right]$$

(45) $$P_{B}=P_{C}(1-P_{A})/2$$

(46) e=1−(BA)2

where R is the aspect ratio of the nanorods (width/length).

For a nanomatryushka (NM), the absorption cross-section is significantly influenced by the volumetric contributions of its multiple concentric layers and is expressed as [36].

(47) Cabs=2∫NMPLSPRdVnmε0μ0|EincT|2

where $P_{\mathrm{LSPR}}$ is the power absorbed by the nanoparticle, $\varepsilon_{0}$ and $\mu_{0}$ are the permittivity and permeability of free space, $n_{m}$ is the refractive index of the surrounding medium, and $E_{\mathrm{inc}}^{T}$ is the transverse component of the incident laser field.

Finite-difference time-domain (FDTD) simulations and discrete dipole approximation (DDA) methods are widely used to model the optical properties of non-spherical plasmonic nanostructures. FDTD discretises both space and time to solve Maxwell’s equations under appropriate boundary conditions, enabling computation of electric and magnetic field distributions throughout the structure [43,44,45,46].

For plasmonic metals such as Au, Ag, and Cu, free-carrier densities typically lie in the range of 10^22^–10^23^ cm^−3^. Most semiconductors do not naturally possess such high carrier concentrations, preventing LSPR excitation in the visible range. However, semiconductor carrier densities can be tuned through doping, analogous to how plasmonic resonance in metals can be tuned by modifying particle size, shape, and dielectric environment. In doped semiconductors operating in the NIR–mid-IR regime, LSPR can be supported when free-carrier concentrations fall within 10^16^–10^19^ cm^−3^ [43].

This overview demonstrates that, both theoretically and experimentally, the formulation of plasmonic characteristics for a given material system has advanced considerably since Gustav Mie’s pioneering solution for light scattering by spherical particles [43,47,48]. Building on the fluorescence-related discussions presented earlier—namely dielectric losses, the wavelength dependence of plasmon excitation, the tunability of resonance through morphological control, the modulation of absorption profiles, the efficiency of plasmonic excitation, and the importance of structural homogeneity—we now turn to how these foundational principles manifest in practice. In the following section, we establish the conceptual and mechanistic links that describe how an optimally excited plasmon can influence, enhance, or modulate the fluorescence response of a given fluorophore.

## 5. Bonding SPR and Fluorescence

Building on the preceding discussions, the domains of fluorescence and surface plasmon resonance (SPR) have been outlined with the aim of identifying areas where synergistic interactions between SPR-active materials and fluorescent systems can be exploited. Such interactions can lead to pronounced modifications in fluorescence characteristics, including lifetime, quantum yield, emission intensity, and even material stability. To structure this discussion, the present section is divided into two parts: Section 5.1 highlights the specific attributes of SPR and fluorescence that enable meaningful overlap, while Section 5.2 examines mechanistic insights from experimental and theoretical studies that have explored—and in many cases confirmed—the nature of these plasmon–fluorophore connections.

### 5.1. Theory and Aspects of Plasmons Relevant to Fluorescence

Modifications to fluorescence in the presence of plasmonic nanostructures arise from several key mechanisms:

(1) the lightning-rod effect associated with localized surface plasmons,
(2) radiative and non-radiative coupling pathways,
(3) near-field and far-field electromagnetic interactions, and
(4) energetic correspondence between plasmon resonances and fluorophore transitions.

The localized electromagnetic field generated by LSPR is typically the dominant contributor to fluorescence enhancement. By concentrating the incident field into sub-wavelength volumes, LSPR can significantly increase a fluorophore’s quantum efficiency and photostability, thereby amplifying its observable emission [2].

A second major factor is the coupling between surface plasmons and fluorophores, mediated by both radiative and non-radiative interactions. This coupling depends strongly on the spectral overlap between the plasmon resonance and the fluorophore’s absorption band, and can lead to either fluorescence enhancement or quenching. Non-radiative energy transfer is governed not only by the strength of the local electromagnetic field but also by the degree of spectral overlap, as demonstrated in recent theoretical and experimental studies. Fluorescence enhancement at separations of ~10 nm is often attributed to Förster resonance energy transfer (FRET), whereas the Purcell effect accounts for enhancements observed at larger distances (10–50 nm). In essence, plasmonic nanostructures enhance fluorescence by harvesting light across broad spectral ranges inaccessible to pristine fluorophores and by confining electromagnetic energy within nanoscale regions that fluorophores can occupy [49,50,51].

**Figure 8:**
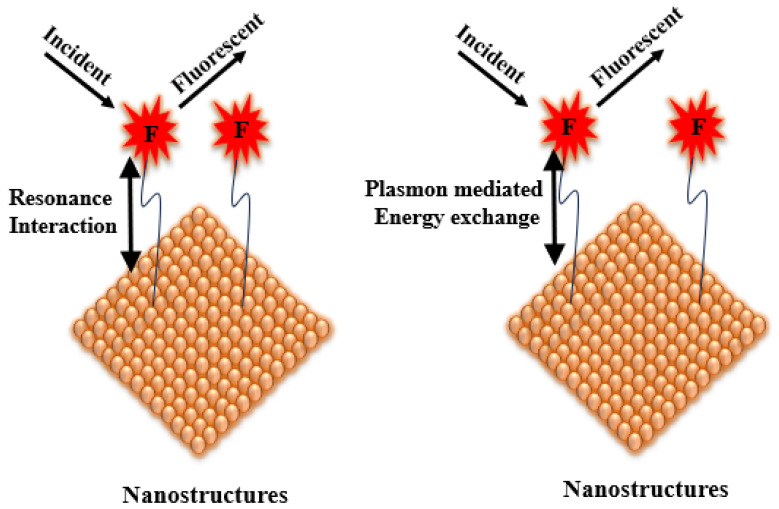
Early interpretation of metal-enhanced fluorescence (**Left**), in which fluorophores experience a modified radiative decay rate in the presence of a metallic surface. The revised interpretation (**Right**) incorporates the contribution of the plasmonic system itself, wherein coupled plasmon–fluorophore interactions lead to additional emission pathways and enhanced overall fluorescence output [54].

A third factor involves modifications to the intrinsic fluorescence lifetime when fluorophores are positioned near plasmonic nanostructures. New MMF decay pathways emerge due to plasmon–fluorophore coupling. For example, increased non-radiative energy transfer from the metal to the fluorophore can result in enhanced radiative emission into the far-field, thereby increasing fluorescence intensity. By tailoring the geometry and composition of plasmonic structures, one can tune the radiative decay rate of the fluorophore. This accelerated radiative decay manifests as a reduced fluorescence lifetime. The balance between plasmon absorption and scattering cross-sections—strongly dependent on nanoparticle size—is a useful predictor of MMF behaviour: absorption dominates for particles smaller than ~20 nm, whereas scattering becomes increasingly significant for larger structures. This size-dependent balance is therefore a valuable guide for anticipating changes in fluorescence lifetime and related MMF parameters [2,52,53]. A generalised schematic of the evolution in the mechanistic understanding in MMF research is presented in Figure 8.

### 5.2. Mechanisms of MMF

Metal-Manipulated Fluorescence (MMF) refers to the deliberate modulation of luminescence using metallic nanostructures such as Ag, Au, or Cu. These plasmonic materials alter the natural fluorescence behaviour of nearby fluorophores, influencing key attributes including emission intensity, fluorescence lifetime, quantum yield, and photostability. At its core, MMF arises from the interplay between enhanced electromagnetic fields generated by plasmonic resonances and modifications to the intrinsic photophysical pathways of the fluorophore.

Because fluorescence may be either enhanced or quenched depending on the geometry, composition, and separation distance between the fluorophore and the plasmonic structure, understanding the mechanisms of energy exchange between the two becomes essential. In the following subsections, we disentangle these mechanisms, examining how plasmonic near-fields, radiative and non-radiative coupling pathways, and resonance matching collectively govern the MMF response.

The discussion in this review is restricted to Fluorescence enhancement, but even more enhancements to emission are possible, for example through enhancing phosphorescence [55] wherein reports show definitively that plasmonic nanostructures could affect radiative and non-radiative decays of emitters considerably. Improvements to plasmonic nanostructure configuration have allowed more efficient interactions of light with matter, expanding the applications of plasmon-mediated emission enhancement. Localized plasmonic nanocavities exhibit strongly inhomogeneous local electric fields due to strong plasmonic field concentration. This leads to strong plasmon-induced magnetic fields, a novel phenomenon that cannot be encountered under classical optical excitations. These strong magnetic fields even allow spin selection rules to be overcome, based on recent theoretical and experimental reports. This allows spin-forbidden singlet/triplet inter-system crossing to be directly activated, enabling the efficient population of triplet states, even in cases of low spin–orbit coupling. With such a level of plasmonic confinement, the intensities of the triplet emission may even surpass those of the singlet emission. Indeed, such enhancements have been reported even in the absence of the nanocavities, wherein a layered structure, as detailed in Section 6.1, results in strong optomagnetic confinement and fluorescent enhancement [56].

**Figure 9:**
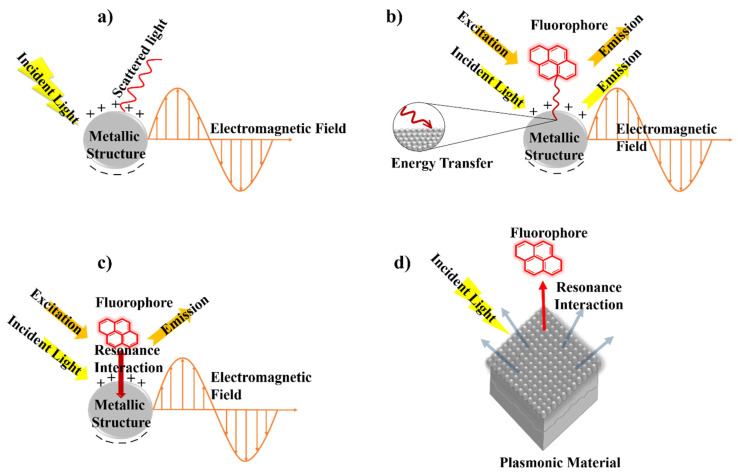
Schematic overview of key mechanisms underlying metal-manipulated fluorescence (MMF): (**a**) localized surface plasmon resonance (LSPR) effects arising from the metallic nanostructure; (**b**) plasmon coupling mediated by radiative and non-radiative interactions between adjacent nanoparticles or between a nanoparticle and a fluorophore; (**c**) modification of the intrinsic radiative decay rate of the fluorophore in the presence of a metal; and (**d**) plasmon-induced fluorescence enhancement on a nanostructured metallic substrate.

As illustrated in Figure 9, the primary driver of enhanced fluorophore excitation in MMF systems is the intensified electromagnetic field generated near the metal nanostructure. Because plasmon lifetimes are considerably shorter than typical fluorescence lifetimes, the local electric field surrounding the metal is strongly amplified, thereby increasing the excitation rate of nearby fluorophores. The magnitude of this enhancement depends on several factors, including the metal type, nanoparticle size and shape, and the excitation wavelength.

**Figure 10:**
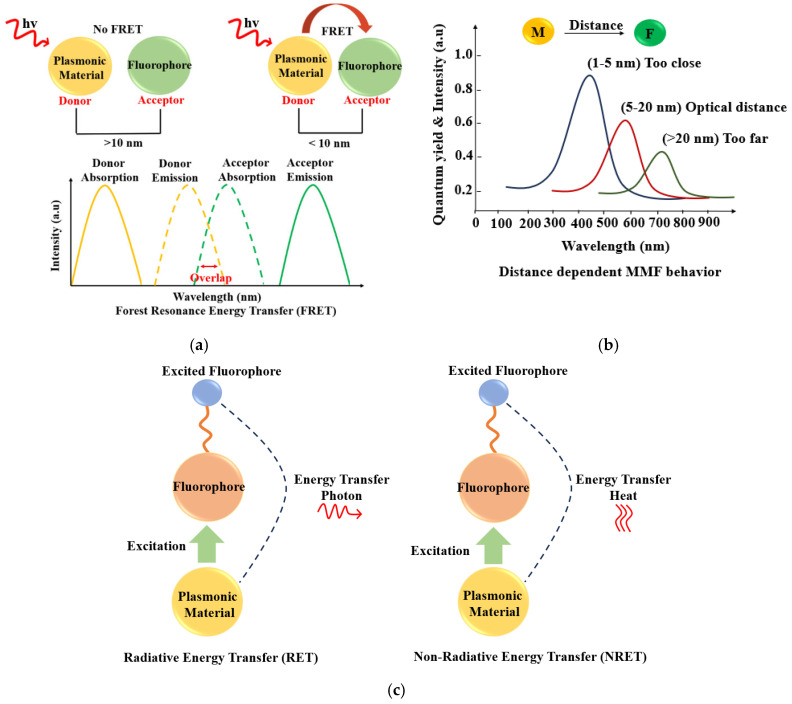
Schematic representations of key interaction pathways between plasmonic structures and fluorophores: (**a**) Förster resonance energy transfer (FRET) mechanism between a plasmonic donor and a fluorophore acceptor; (**b**) distance-dependent MMF behavior illustrating the transition from quenching at very short separations to optimal enhancement at intermediate distances; (**c**) radiative and non-radiative energy-transfer channels from the plasmonic structure to the fluorophore, highlighting how these pathways collectively influence emission intensity and lifetime.

In addition to modifying excitation, plasmonic structures alter the radiative decay rate of fluorophores by introducing additional decay channels. This accelerated return of excited electrons to the ground state leads to brighter emission and shorter fluorescence lifetimes in time-resolved measurements. Among all parameters governing MMF, the fluorophore–metal separation distance is one of the most critical. At very short distances (<5 nm), non-radiative energy transfer mechanisms—such as surface energy transfer or Förster resonance energy transfer (FRET, depicted in Figure 10)—dominate, resulting in fluorescence quenching. At intermediate distances (5–20 nm), optimal coupling between the plasmonic near-field and the excited fluorophore produces fluorescence enhancement. Beyond ~30 nm, the influence of the metal diminishes substantially, and the fluorophore behaves largely as it would in the absence of the plasmonic structure.

Extensive investigations into MMF—and specifically metal-enhanced fluorescence (MEF)—have established three principal interaction pathways. The first is excitation enhancement, in which the fluorophore’s effective absorption cross-section increases due to the intensified local electromagnetic field generated by the metal nanoparticle. Under identical incident power, this leads to more efficient excitation and consequently a greater number of emitted photons.

The second pathway is emission coupling, where the excited fluorophore couples to plasmon modes on the metal surface. This coupling facilitates more efficient radiation of energy into the far-field, often producing emission that is both polarized and directional. The third pathway involves dipole–dipole interactions, enabling non-radiative energy transfer from the fluorophore to the metal when the separation distance is extremely small. In this regime, the fluorophore is too close to the metal surface, resulting in energy dissipation as heat and a corresponding reduction in fluorescence lifetime.

Multiple factors influence MMF performance, including metal type, nanostructure geometry, fluorophore characteristics, spacer layer properties, dipole orientation, and surface coverage. Metal choice is particularly critical: Ag typically provides the highest enhancement due to its sharp plasmon resonance in the visible region, whereas Au offers superior chemical stability and biocompatibility but generally yields lower enhancement. Nanostructure geometry—such as nanorods, nanoshells, nanostars, and core–shell architectures—strongly affects resonance conditions, electromagnetic field distributions, and the tunability of plasmonic properties.

Because direct metal–fluorophore contact leads to quenching, spacer layers such as silica or polyvinyl alcohol are often employed to maintain an optimal separation distance that favours enhancement over quenching. Fluorescence parameters—including lifetime, quantum yield, emission intensity, and absorption/emission characteristics—are all governed by the strength and spatial distribution of the metal-enhanced field. Additionally, fluorophore dipole orientation and surface coverage play important roles: controlled dipole alignment and appropriate fluorophore density can promote enhancement, whereas excessive loading may induce self-quenching and energy losses.

**Figure 11:**
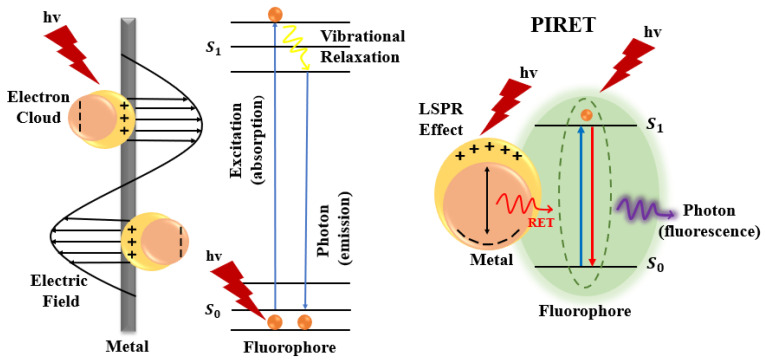
Schematic illustration of plasmon-induced resonance energy transfer (PIRET). In this mechanism, energy is transferred non-radiatively from an excited plasmonic nanoparticle to a nearby fluorophore through dipole–dipole coupling, enabling enhanced excitation without requiring direct photon emission from the metal.

Optimizing these parameters is essential for maximizing MMF performance. The combined influence of plasmonic attributes on fluorescence behaviour is illustrated in Figure 11.

Up to this point, we have outlined the foundational concepts underlying MMF by examining fluorescence behaviour, the two principal plasmonic mechanisms that modulate fluorescence, and the direct physical connections between plasmon resonances and fluorophore responses. We now turn to specific studies that report how fluorescent molecules and nanostructures behave when placed in proximity to plasmonic materials. This investigation will compare a range of characteristics to understand how plasmonic attributes manifest in different fluorescent systems. While changes in fluorescence lifetime, quantum yield, and emission intensity remain the most common evaluative parameters, our primary emphasis will be on factors such as nanomaterial composition, morphology, modality (film versus particle), and the wavelength of plasmonic absorption. These parameters will be examined to determine whether particular material characteristics correlate with predictable fluorescence modulation, with the broader goal of identifying the most effective plasmonic material for a given experimental context—whether defined by synthesis method, fluorophore type, or structural morphology—while also remaining attentive to potential adverse or deteriorative effects that may arise at the boundaries of fluorescence–plasmon interactions.

## 6. MMF-Exploring Plasmonic Interactions for Enhanced Optical Properties

In summarizing the potential of MMF within this review, our focus now shifts to how different synthesis and fabrication strategies enable the realization of plasmonically tuned fluorescent structures. Central to this discussion are the major quantifiable fluorescence parameters—lifetime, quantum yield, and emission intensity—which serve as the primary indicators of plasmon-induced modulation. To maximize the interaction between metal nanostructures and fluorescent materials, a wide range of fabrication and coating techniques has been developed, each offering distinct advantages in controlling spatial arrangement, separation distance, and structural uniformity.

This section examines several widely used approaches for integrating plasmonic materials with fluorophores, including Sandwich coating (Section 6.1), Spin coating (Section 6.2), Layer-by-Layer assembly (Section 6.3), the Langmuir–Blodgett technique (Section 6.4), Dip coating (Section 6.5), Self-Assembled Monolayers (Section 6.6), Electroless Deposition (Section 6.7), Sputter Coating (Section 6.8), Photolithography (Section 6.9), and Electron Beam Lithography (Section 6.10). In order to have an easily viewable comprehension of the presented data, we present the findings from the individual reports in two formats viz. the first being a representation through table of the principal challenges that were observed in each study. The second is a summary figure, which includes details on each material used in the different layers/locations of a particular synthesis procedure, a summary on the enhancements observed in the fluorescence properties and a summary on the different linker molecules used to couple the plasmonic structure/material with the fluorescent entity. Together, these methods provide a versatile toolbox for engineering plasmon–fluorophore architectures with enhanced optical performance.

### 6.1. Sandwich Coating

A sandwich coating is a multilayer deposition strategy designed to enhance the performance, stability, and optical functionality of material surfaces. Typically, such systems consist of a priming layer to promote adhesion, an intermediate functional layer that imparts specific properties (e.g., corrosion resistance, plasmonic activity, fluorescence modulation), and a protective topcoat that stabilizes the structure and improves durability. By engineering light–matter interactions at the nanoscale, sandwich coatings can be tailored to enhance fluorescence, improve photostability, and increase detection sensitivity in optical and sensing applications [57,58,59]. In this section, we highlight representative studies and summarize broader trends to maintain clarity and avoid excessive length.

**Figure 12:**
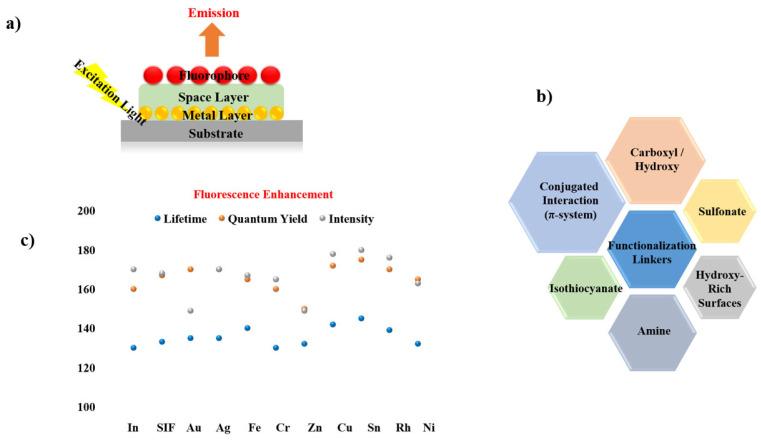
Summary of materials commonly employed in sandwich-type MMF coatings, including (**a**) sandwiched coated MMF structure consisting of substrate, metal layer (Ni [56], Rh [64,66], Sn [57], Cu [57,66], SIFs [17,19,25,63,64,65], Zn [58], Cr [58], In [13], Al, Ag [60], Au [61], Fe [62]), spacer layer (PVA/PMMA [13,17,19,25,56,57,60,62,64,65,67], SiO_2_ [61,63,66], BSA [5,17], oxide [13,57,58,64]), and fluorophore (Pery [13,25], IR792, FITC [65], AO [57,62], RB [19,58], Rh101, NB [56],FL [17,57,58,60], CBS [13,64,67], IR780, Blood [17], 7HC, BF, ICG [58], SWNTs [61], SiO_2_ [63], DCM [64], Rh800 [66]). (**b**) Functional linkers (Conjugated Interaction [17,25,56,61,64,66], Carboxyl/Hydroxy [17,19,57,58,60], Amine [17,57,58,62], Sulfonate [13,58,64], Hydroxy-Rich Surfaces [17,63], Isothiocyanate [65]) chemistries employed for fluorophore immobilization on metal surfaces. (**c**) Comparison of fluorescence intensity, quantum yield, and lifetime for different metal systems.

**Figure 13:**
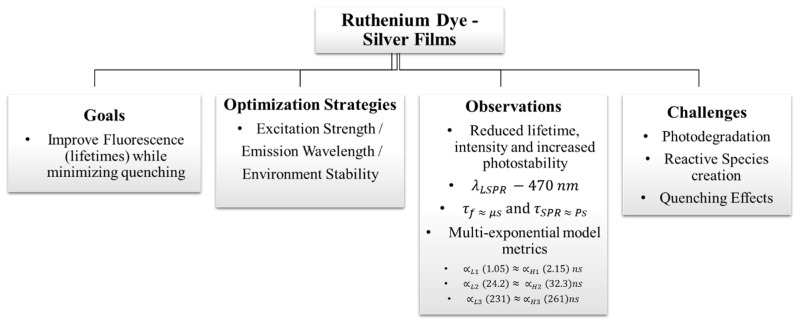
Enhancement of metal-manipulated fluorescence (MMF) through optimized interactions between a ruthenium dye and a silver (Ag) film. The figure illustrates how controlled fluorophore–metal spacing and tailored film morphology improve excitation efficiency and emission intensity, demonstrating the tunability of MMF performance in dye–metal hybrid systems [12].

**Figure 14:**
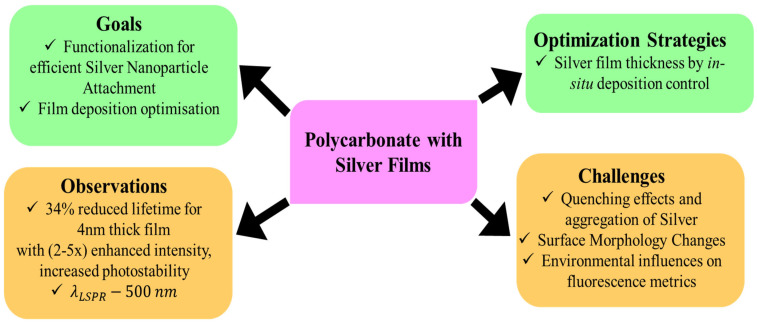
Optimization of interactions between polycarbonate substrates and silver (Ag) films to maximize fluorescence efficiency in metal-manipulated fluorescence (MMF). The figure illustrates how controlled film deposition, surface morphology, and fluorophore–metal spacing collectively influence enhancement performance in polymer–metal hybrid platforms [70].

Given the widespread use of sandwich coating across MMF studies, the remaining reports are summarized through graphical illustrations Figure 12 and a concise tabulation in Table 1, with selected studies highlighted in Figure 13 and Figure 14.

**Table 1:** Summary of sandwich-type MMF coatings, including experimental challenges with corresponding literature references.

S.No	Challenges	Ref.
1.	Material stability Photostability inferior performance to metals Integration into existing technologies	[13]
2.	Complexity of interactions Quantitative analysis Reproducibility	[25]
3.	Complexity of interactions remains unresolved Reproducibility Understanding Plasmonic dynamics	[60]
4.	Uniformity of coating Characterization Understanding nonradiative decay	[61]
5.	Uniformity in nanoparticle morphology Measurement accuracy Environmental stability	[62]
6.	Fabrication consistency, material stability Unraveling interaction complexity Measurement sensitivity	[63]
7.	Signal overlap Temporal resolution Interpreting complex dynamics Reproducibility	[64]
8.	Surface Stability Control of Nanoparticle Size Fluorescence collection optimization Long-term stability	[65]
9.	Quantifying enhancement Measurement consistency Corroborations with theoretical models	[19]
10.	Surface stability Quantitative analysis	[66]
11.	Thickness-dependent quenching Intensity–quantum yield trade-off Non-radiative decay control	[67]
12.	Substrate-induced quenching Limited quantum-yield improvement Lifetime reduction in thicker films	[13]
13.	Dominant non-radiative energy transfer Narrow optimal thickness window Enhancement not universal across metrics	[68]
14.	Island aggregation sensitivity Excitation-irradiance dependence Reproducibility issues	[21]
15.	Dominant non-radiative energy transfer Narrow optimal thickness window Enhancement not universal across metrics	[67]
16.	Narrow optimal thickness window Increased quenching outside optimum Balancing exciton–plasmon coupling	[68]
17.	UV-dominated plasmon response Moderate lifetime loss for some dyes Precise ultrathin thickness control required	[69]

### 6.2. Spin Coating

Spin coating is a widely used thin-film fabrication technique consisting of four sequential stages: deposition, spin-up, spin-off, and evaporation. Among these, the flow-controlled and evaporation-controlled spin-off stages play a decisive role in determining the final film thickness and uniformity. In the final evaporation step, solvent removal becomes critical; longer evaporation times increase the solution concentration and viscosity, ultimately producing thicker films. This parameter must therefore be carefully monitored and controlled—often through the application of heat—to achieve the desired coating characteristics [71,72].

**Figure 15:**
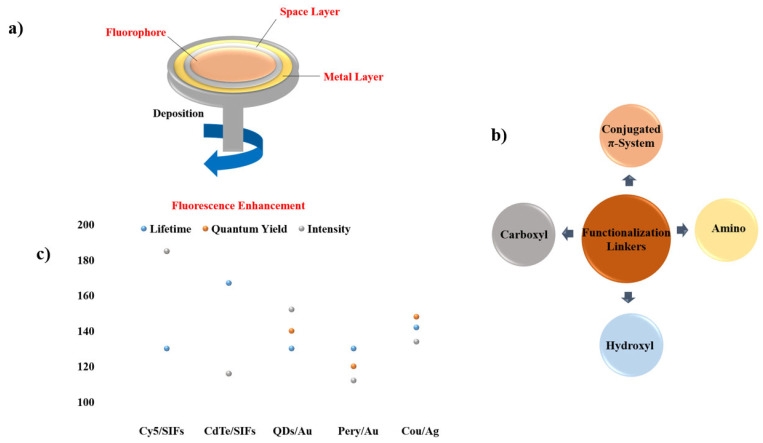
Summary of materials employed in MMF studies using the spin-coating synthesis approach. (**a**) Spin-coated MMF structure consisting of substrate, metal layer (SIFs [73,74], Au [75,76,77], Ag [77]), space layer (PMMA [74,76], PEG [77], Polycarbonate [75], SiO_2_ [73]), and fluorophore (Cy5 [73], CdTe [74], QDs [75], Pery [76], Cou [77]). (**b**) Functional linkers (Conjugated π-System [76], Amino [73], Hydroxyl [77], Carboxyl [74]) chemistries employed for fluorophore immobilization on metal surfaces. (**c**) Comparison of fluorescence intensity, quantum yield, and lifetime for different metal systems.

A wide range of fluorescent compounds and their plasmonic counterparts have been fabricated and studied using spin coating, enabling systematic evaluation of MMF behaviour under controlled film-formation conditions. Technical insights from these studies, including material combinations, film morphologies, and resulting fluorescence modifications, are summarized in Figure 15 and with the key details presented concisely in Table 2.

**Table 2:** Summary of the experimental challenges with associated literature references for spin-coated sandwich-type MMF systems.

S.No	Challenges	Ref.
1	Uniform coating Fluorophore stability Background noise	[73]
2	Complex Interactions Variability in measurements Scaling studies	[74]
3	Optimizing coating thickness Scalability Balancing performance with cost Understanding long-term stability	[75]
4	Achieving optimal spacing Control over nanoparticle distribution Scalability Optimization of polymer matrices	[76]
5	Optimization of nanoparticle distribution Scalability Thermal stability Design as thin film	[77]

### 6.3. Layer-by-Layer Coating

Layer-by-layer (LBL) coating is a versatile assembly technique in which a charged substrate is alternately exposed to solutions containing positively and negatively charged polyelectrolytes. After each adsorption step, the substrate is rinsed—typically with purified water—to remove excess polyelectrolytes and prevent cross-contamination between solutions, followed by drying. This sequential deposition enables precise molecular-level control over film roughness, thickness, and porosity by adjusting parameters such as pH, ionic strength, and polyelectrolyte concentration.

**Figure 16:**
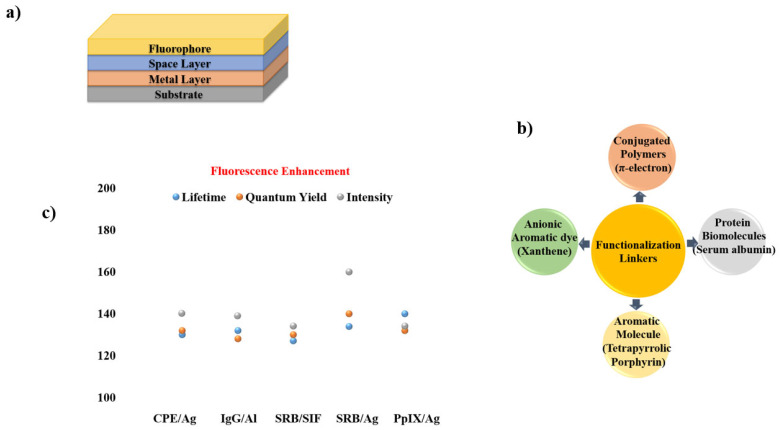
Summary of materials employed in MMF studies using the Layer-by-layer coating. (**a**) Layer-by-layer coating MMF structure consisting of substrate, metal layer (Ag [83,84,85], Al [82], SIF [83]), space layer (PAH [21,81], BAS [82], SiO_2_ [83,85]), and fluorophore (CPE [81], BSA/IgG [82], SRB [83,84], R123 [84], PpIX [85]). (**b**) Functional linkers (Conjugated Polymers (π-electron) [81], Protein Biomolecules (Serum albumin) [82], Aromatic Molecule (Tetrapyrrolic Porphyrin) [83], Anionic Aromatic dye (Xanthene) [83]) chemistries employed for fluorophore immobilization on metal surfaces. Comparison of fluorescence intensity, quantum yield, and lifetime for different metal systems. (**c**) Comparison of fluorescence intensity, quantum yield and lifetime for different metal systems.

Although LBL assembly is fundamentally driven by electrostatic attraction between oppositely charged species, multilayer growth can be further stabilized or enhanced through additional interactions, including covalent bonding, hydrogen bonding, charge-transfer interactions, hydrophobic forces, and coordination bonding. These diverse interaction pathways allow the fabrication of highly tunable architectures suitable for MMF applications. A range of such LBL-based plasmonic–fluorophore structures reported in the literature [78,79,80] is summarized in Figure 16 and with the key details presented concisely in Table 3.

**Table 3:** Summary of materials developed for MMF using layer-by-layer assembly, outlining the principal challenges associated with this approach.

S.No	Challenges	Ref.
1	Fabrication reproducibility Consistent sensitivity Surface functionalization and stability	[81]
2	Distance control Protein denaturation and aggregation Fluorescence lifetime measurements Surface Functionalization	[82]
3	Fluorophore attachment consistency Polarized absorption spectroscopy limitations Fluorescence lifetime measurement accuracy Controlling fluorophore probe interactions	[83]
4	Dye aggregation control, selectivity Reproducibility of signal enhancement Surface stability and longevity Miniaturization and portability	[84]
5	Precise control of polyelectrolyte layer thickness Nanoparticle aggregation or clustering Fluorophores quenching Reproducibility Of the system Fluorescence signal saturation	[85]

### 6.4. The Langmuir-Blodgett (LB) Method

The Langmuir–Blodgett (LB) technique has been widely employed in surface-enhanced fluorescence (SEF) research due to its exceptional ability to control film architecture, thickness, and surface homogeneity. It remains one of the most effective methods for preparing and characterizing ordered molecular assemblies, particularly ultrathin solid films. Both surface coverage and intermolecular spacing can be precisely tuned, and the latter—critical for SEF studies—can be adjusted using fatty-acid-based LB spacer layers to regulate the distance between fluorescent molecules and plasmonic nanostructures.

**Figure 17:**
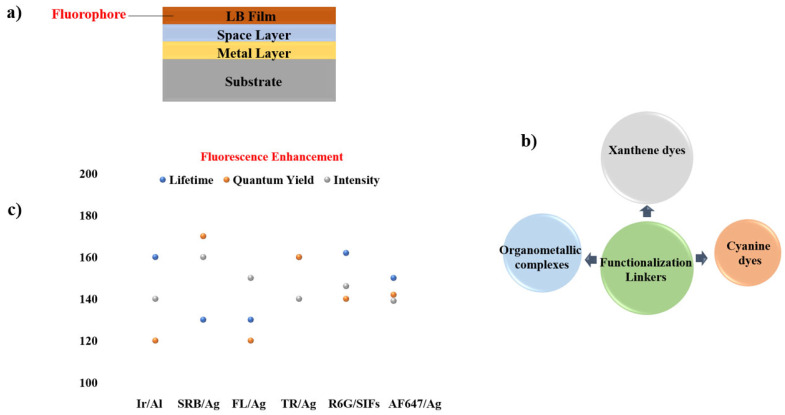
Summary of materials employed in MMF studies using the Langmuir–Blodgett deposition technique. (**a**) Langmuir–Blodgett method MMF structure consisting of substrate, metal layer (Al [89], Ag [89,90,91,92,94], Au [92], SIFs [93]), spacer layer (PMMA [89,90], Al_2_O_3_ [89], SiO_2_ [90,91,93], LB films [90], PEG [90], SAMs [90,92,93,94]), and fluorophore (Ir(III) [89], SRB [90], TR [92], R6G [93], FITC [91],AF64 [94]). (**b**) Functional linkers (Xanthene dyes [90,91,92], Cyanine dyes [93], Organometallic complexes [89]) chemistries employed for fluorophore immobilization on metal surfaces. (**c**) Comparison of fluorescence intensity, quantum yield, and lifetime for different metal systems.

This precise control over nanoscale spacing enables systematic investigation of the distance dependence of SEF, allowing fluorescence intensities to be maximized by minimizing non-radiative energy transfer to the metal. As demonstrated in multiple studies [86,87,88] optimizing this separation distance is essential for achieving strong enhancement while avoiding quenching. The data summarized in Figure 17 highlight how fluorescence intensity and related parameters vary under different LB-controlled conditions, underscoring the importance of nanostructure–molecule spacing in determining overall MMF performance and the key findings are presented concisely in Table 4.

**Table 4:** Summary of the MMF materials fabricated by Langmuir–Blodgett deposition, emphasizing key experimental challenges and associated literature references.

S.No	Challenges	Ref.
1	Metal absorption losses Hard to differentiate plasmonic enhancement from interference effects. Thickness uniformity	[89]
2	Challenging to separate the contributions of radiative and non-radiative decay processes to the changes in lifetime Control of environmental factors	[90]
3	Instability of multilayers Self-quenching effects Reproducibility	[91]
4	Reproducibility issues Nanoparticle stability Environmental sensitivity	[92]
5	Surface coverage effects and aggregation Temperature sensitivity Reproducibility	[93]
6	Spectral overlap issues Complex fabrication process of klarite coating with Ag and silica	[94]

### 6.5. Dip Coating

Dip coating is a simple, cost-effective, and widely adopted technique for depositing thin films onto a variety of substrates, including fibres, ceramics, metals, and polymers. In this process, the substrate is immersed in an aqueous-based coating solution, withdrawn at a controlled rate, and subsequently dried. During withdrawal, a wet film forms on the substrate surface; upon solvent evaporation, this film solidifies into a uniform dry coating. Because the target materials are deposited in situ from solution, dip coating offers a straightforward route to fabricating plasmonic–fluorophore architectures with tunable thickness and composition [95].

**Figure 18:**
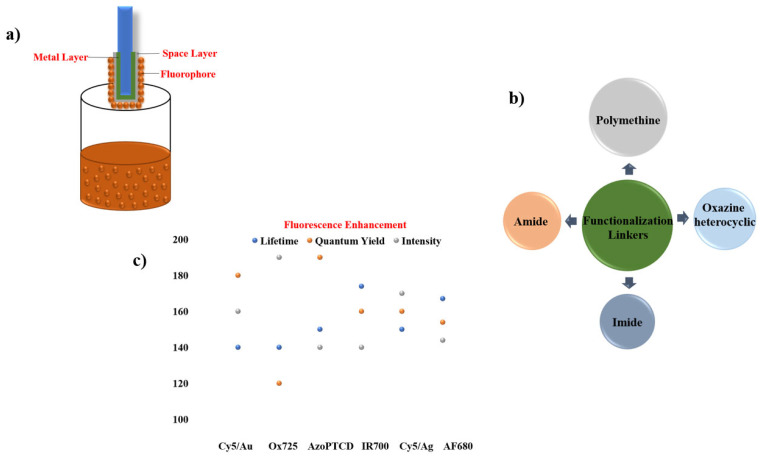
Summary of materials employed in metal-manipulated fluorescence MMF studies using the Dip coating. (**a**) Dip coating method MMF structure consisting of substrate, metal layer (Ag [96,101], Au [97,100], Cu [98], SIFs [99]), spacer layer (PMMA [96], PVA [96], Polycarbonate [97], SiO_2_ [98,99], Alkanethiols [100]), and fluorophore (Cy5 [96], Ox725 [97], AzoPTCD [98], IRDye800CW/IRDye700 [99], Cy3 [100], AF680 [101]) (**b**) Functional linkers chemistries employed for fluorophore immobilization on metal surfaces. (**c**) Comparison of fluorescence intensity, quantum yield, and lifetime for different metal systems.

A range of MMF studies employing dip-coated structures has been reported, demonstrating how this technique can modulate fluorescence through controlled film formation, metal–fluorophore spacing, and nanoparticle distribution. Key findings from these investigations are summarized in Figure 18 with the details presented concisely in Table 5.

**Table 5:** Summary of materials developed for MMF via dip-coating, with a focus on key experimental challenges.

S.No	Challenges	Ref.
1	Uniform coating and layer integrity for preventing defects Control of metal thickness and size variability	[96]
2	Uniformity in coating Characterization difficulties on nanorod orientation and on the impacts of various parameters	[97]
3	Oxidation of Cu can reduce plasmonic enhancement Grain boundary defects lead to increased SEF signal variations Complex fabrication required precise nanosphere coating and electrodeposition control	[98]
4	Controlling SIF uniformity and nanoparticle size distribution Reducing unwanted background signals from Ag nanoparticles	[99]
5	Balancing fluorescence enhancement while avoiding quenching Reducing background fluorescence Maintaining uniformity in dip-coated PEM films	[100]
6	Lack of precise control over metal island size and distribution Reproducibility of SIFs was difficult due to randomized deposition Background scattering affected low-concentration detection	[101]

### 6.6. Self-Assembled Monolayers (SAMs)

Self-assembled monolayers (SAMs) are highly ordered molecular assemblies that form spontaneously when long-chain molecules chemisorb onto solid substrates. Among the most extensively studied systems are alkanethiol SAMs on gold, driven by the strong affinity of sulfur for Au surfaces. These structures are widely employed in microfabrication, biosensing, and surface engineering due to their robustness, tunability, and chemical stability. Beyond gold, SAMs can be formed on a variety of substrates—including Ag, Cu, Pt, Hg, GaAs, and InP—making them broadly applicable across plasmonic and electronic platforms.

SAMs can be fabricated through several routes, including vapor-phase deposition, potential-controlled adsorption, or immersion of substrates into thiol-containing solutions. These methods offer excellent adaptability and allow precise control over surface properties such as wettability, charge density, and ionization behaviour. Importantly, SAMs enable stable and predictable functionalization without compromising structural integrity, making them particularly valuable for MMF applications [102,103,104].

**Figure 19:**
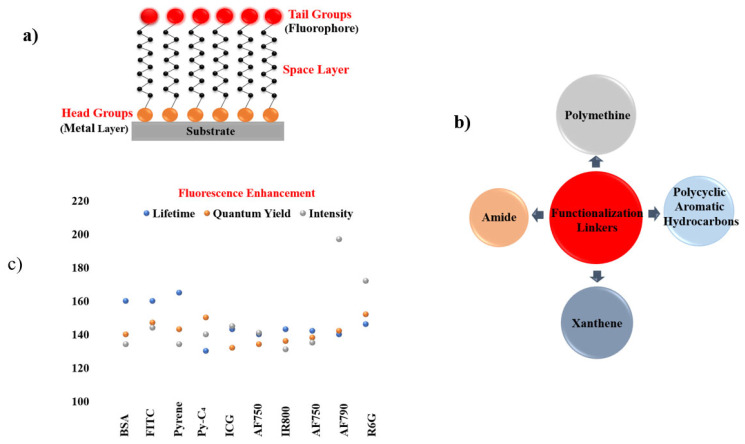
Summary of materials fabricated as self-assembled monolayers for MMF studies. (**a**) SAMs method MMF structure consisting of substrate, metal layer (Au [105,108,109,110,111,112], Ag [91,113], SiO_2_ [106], Al [107]), space layer (BAS/HSA [105], Silica [108,109,110], Polymer [105,108,113], SAM [91,113], Alkyl chain [111,112]), and fluorophore (HSA, BSA [105], FITC, Biotin-FITC, NBD-RagIgG [91], Pyrene [106], Py-C_12_/Py-C_16_/Py-C_4_ [107], ICG [108], AF750, AF790 [109], IRDye-800CW [110], AF750 [111], AF790 [112], R6G [113]). (**b**) Functional linkers (Polymethine [108,110,111,112], Polycyclic Aromatic Hydrocarbons [106,107], Xanthene [91,113], Amide [91,105]) chemistries employed for fluorophore immobilization on metal surfaces. (**c**) Comparison of fluorescence intensity, quantum yield, and lifetime for different metal systems.

The fluorophores incorporated into SAM-based MMF systems exhibit distinct absorption and emission characteristics depending on molecular structure, surface chemistry, and metal–fluorophore spacing. Figure 19 summarizes the key optical properties of fluorophores utilized in SAM architectures and highlights how SAM-mediated organization influences their MMF behaviour, with the details presented concisely in Table 6.

**Table 6:** Summary of Key Features for SAM-based MMF Materials with Emphasis on Important Experimental Challenges and their Associated Literature References.

S.No	Challenges	Ref.
1	Reproducibility in the self-assembly processes to maintain uniform enhancement across the substrate	[105]
2	Reproducibility in assembling uniform SAMs Aggregation of Ag nanoparticles	[91]
3	Fluorescence quenching due to non-radiative energy transfer Reproducibility issues in SAMs Optimizing spacer chain length	[106]
4	Low pyrene concentration makes detection via FTIR difficult Fluorescence signal interference due to metal substrate proximity Difficulty in directly measuring probe localization within the monolayer	[107]
5	Quenching effects Reproducibility Requires complex electromagnetic modelling and exact fabrication methods	[108]
6	Difficulty in precisely controlling the morphology of nanoparticles. Biocompatibility	[109]
7	Sensitivity to environmental factors Material integration impacts on fluorescence.	[110]
8	Reproducibility Avoiding fluorescence from being quenched for short fluorophore-metal distances	[111]
9	Long-term stability is limited by fluorophore photo bleaching. Uncontrolled synthesis of Au nanoparticle formation influences enhancement unpredictably	[112]
10	Optimization of distance Fabrication stability Substrate aggregation	[113]

### 6.7. Electroless Deposition (ELD)

Electroless deposition (ELD) is a versatile coating technique in which materials are deposited from aqueous solutions without the need for an external electrical current. Although most commonly associated with the deposition of metals and metal alloys, the method is equally applicable to the formation of oxides, salts, polymers, and other functional coatings. Its ability to uniformly coat complex surfaces and its compatibility with a wide range of substrates make ELD a valuable analogue to the fabrication strategies discussed in earlier sections.

**Figure 20:**
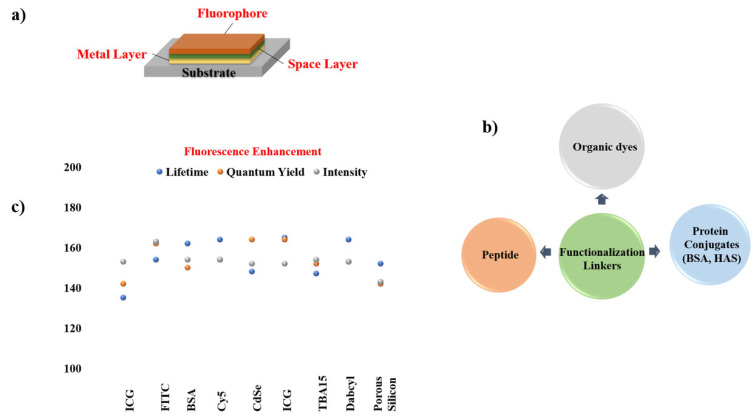
Summary of materials fabricated as Electroless Deposition for MMF studies. (**a**) Electroless Deposition MMF structure consisting of substrate, metal layer (Au [118,120,121,124], Ag [117,119,120,123,124], Ni [125]), space layer (PVA [118,121,122], PMMA [118], PEG [120], SiO_2_ [118,119,125], Polymer [120,123], SAM [123], Ligand shell [121]), and fluorophore (ICG [117,122], GBP-FITC [118,122], BSA [119], Hex [120], CdSe–ZnS QDs [121], TBA15-Cy5 [120,123], FAM/Dabcyl [124], Porous Silicon [125]). (**b**) Functional linkers (Organic dyes [117,120,122,123,124], Protein Conjugates (BSA, HAS) [119,122], Peptide [118,120,122,123]) chemistries employed for fluorophore immobilization on metal surfaces. (**c**) Comparison of fluorescence intensity, quantum yield, and lifetime for different metal systems.

In the context of MMF, electroless deposition offers a controlled and scalable route for integrating plasmonic materials with fluorophores, enabling systematic modulation of fluorescence through tailored film thickness, nanoparticle distribution, and metal–fluorophore spacing [114,115,116]. Representative MMF studies employing ELD-based architectures, along with their key optical outcomes, are summarized in Figure 20, with the details presented concisely in Table 7.

**Table 7:** Summary of materials developed for MMF using electroless deposition, highlighting the most important experimental difficulties and literature references.

S.No	Challenges	Ref.
1	Ag deposition control Surface uniformity	[117]
2	Reproducibility Requirement of precise nanofabrication methods to regulate the surface characteristics and distribution of nanoparticles.	[118]
3	Non-uniform growth of Ag Difficulty in directly inducing and detecting plasmon resonance because of the significant absorption by silicon in that spectral range.	[119]
4	Surface uniformity & reproducibility Stringent instrumentation requirements due to photo bleaching, dual-mode detection requires SERS and fluorescence microscopy apparatus.	[120]
5	Polymer swelling brought on by solvent exposure marginally changed the heights of NP deposition. Contamination of the surface on delicate silicon nitride membranes Non-specific NP adsorption	[121]
6	Surface roughness control Reproducibility	[122]
7	Long-term measurements on human serum samples showed a small decrease in signal, although the LOD was still clinically relevant. Nanostructure stability	[123]
8	Nanoparticle uniformity Fluorophore distance control, as even though MMF was optimized by the SiO_2_ shell (~8 nm), slight variations occurred which decreased the enhancement.	[124]
9	Structural stability Non-radiative loss Fluorescence efficiency was decreased by quenching effects caused by excess Ni.	[125]

### 6.8. Sputter Coating

Sputter coating is a vacuum-based physical vapor deposition technique capable of producing thin films with excellent uniformity, adhesion, and compositional control. The process typically involves several sequential steps: coating—where materials such as titanium, zirconium, or chromium nitrides and oxides are sputtered onto the substrate; etching—where plasma cleaning enhances surface adhesion; ramp-up—during which the system is heated, and the chamber pressure is reduced; and ramp-down—where controlled cooling protects the integrity of the deposited film. Optimizing the deposition rate is essential, as it improves productivity, reduces operational costs, and enhances industrial scalability and competitiveness [126,127,128].

**Figure 21:**
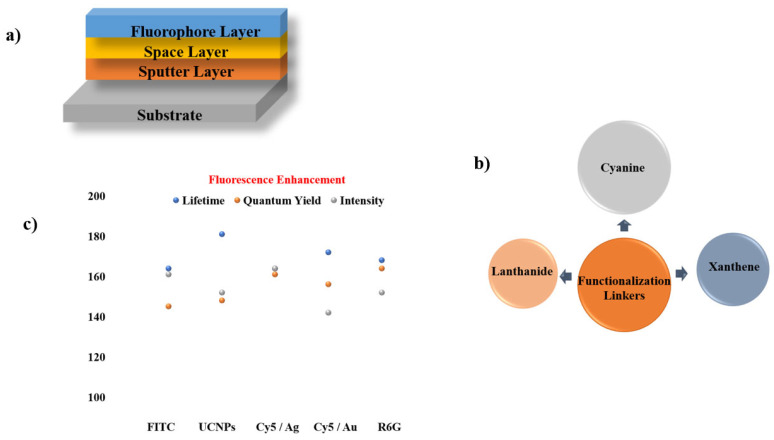
Summary of materials fabricated as Sputter coating for MMF studies. (**a**) Sputter coating method MMF structure consisting of substrate, metal layer (Au [130,132,133], Ag [57,129,131], Cu [133], Sn [133]), space layer (SiO_2_ [129,130,131,132], Avidin monolayer [131], Polymer [133]), and fluorophore (FITC [128], UCNPs (NaYF_4_:Yb,Er) [130], Cy5 [131,132], R6G, Cy3 [133]). (**b**) Functional linkers (Cyanine [131,132,133], Xanthene [129,133], Lanthanide [130]) chemistries employed for fluorophore immobilization on metal surfaces. (**c**) Comparison of fluorescence intensity, quantum yield, and lifetime for different metal systems.

In the context of MMF, sputter coating provides a robust and reproducible method for integrating plasmonic materials with fluorophores, enabling precise control over film thickness, nanoparticle density, and metal–fluorophore spacing. Representative studies employing sputter-coated architectures for fluorescence modulation are summarized in Figure 21. with the details presented concisely in Table 8.

**Table 8:** Summary of materials investigated for MMF using sputter coating, highlighting key experimental challenges and associated literature references.

S.No	Challenges	Ref.
1	Reproducibility issues Fluorescence quenching for fluorophore-metal distances less than 10 nm	[129]
2	Fabrication uniformity Thickness control	[130]
3	Surface roughness Fluorescence quenching Reproducibility issues	[131]
4	Difficult to generate consistent aperture arrays over wide areas due to fabrication difficulty. Reproducibility issues	[132]
5	Reproducibility issues Careful development of fluorophore-metal interactions was required to optimize enhancement while preventing energy loss.	[133]

### 6.9. Photolithography

Photolithography is a powerful and widely used technique for fabricating precisely tailored nanoparticle structures, enabling fine control over spacing, multilayer architectures, particle shapes, and feature sizes. When combined with advanced approaches such as electron-beam lithography, this method can achieve angstrom-level precision, allowing researchers to reproducibly tune key plasmonic enhancement parameters. These include the magnitude of local electric-field amplification, the fluorophore–nanoparticle separation distance, and the immobilization density of fluorophores on or near the nanostructure surface.

**Figure 22:**
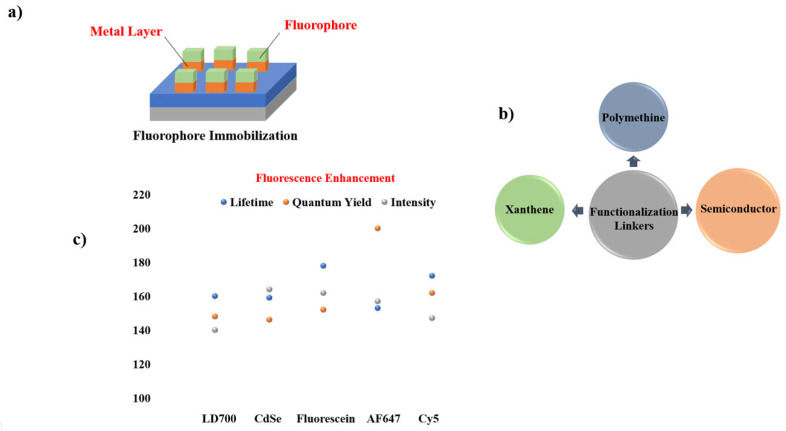
Summary of materials fabricated as Photolithography for MMF studies. (**a**) Photolithography method MMF structure consisting of substrate, metal layer (Au [137,138,139,140], Ag [141]), and fluorophore (LD700 [137], CdSe/ZnS QDs, CdSe QRs [138], Fluorescein [139], AF647 [140], Cy5 [141]). (**b**) Functional linkers (Polymethine [137,141], Semiconductor [138], Xanthene [139,140]) chemistries employed for fluorophore immobilization on metal surfaces. (**c**) Comparison of fluorescence intensity, quantum yield, and lifetime for different metal systems.

Despite its high cost and technical complexity, lithography remains one of the most reliable and indispensable fabrication strategies for achieving optimal fluorescence enhancement through plasmonic effects. Its ability to generate highly ordered, reproducible, and customizable nanostructures makes it particularly valuable for MMF studies where precise spatial control is essential [134,135,136]. Representative investigations employing photolithography-based architectures for fluorescence modulation are summarized in Figure 22, with the details presented concisely in Table 9.

**Table 9:** Summary of developed materials and processes using photolithography for MMF, focusing on the main experimental challenges, and including related literature references.

S.No	Challenges	Ref.
1	Maintaining a consistent dispersion of nanoparticles during micropatterning. Achieving reproducibility in the patterning and fabrication of nanocages.	[137]
2	Managing fluorophore’s distance from the metal to prevent quenching at close range Maintaining the dispersion of nanoparticles in the polymer matrix	[138]
3	Avoiding misalignment in multi-step lithography between nano-patterned features and microfluidic channels	[139]
4	Uniformity in nanoimprinting Preventing electroplating irregularities from causing flaws in the nickel mold production.	[140]
5	Ensuring accurate depth control and reliable repeatability of nanoimprinted gratings. Maintaining consistency in large-area substrate nanoimprinting.	[141]

### 6.10. Electron Beam Lithography (EBL)

Electron beam lithography (EBL) is a high-resolution patterning technique that employs a focused electron beam to generate sub-micron and nanoscale structures on electron-sensitive substrates. When exposed to the electron beam, resist materials undergo characteristic chemical transformations—analogous to the photochemical changes induced by light in conventional photolithography. By precisely controlling the beam position through computer-guided scanning, arbitrary nanoscale patterns can be directly written onto the substrate, enabling faithful transfer of digital designs into physical structures.

**Figure 23:**
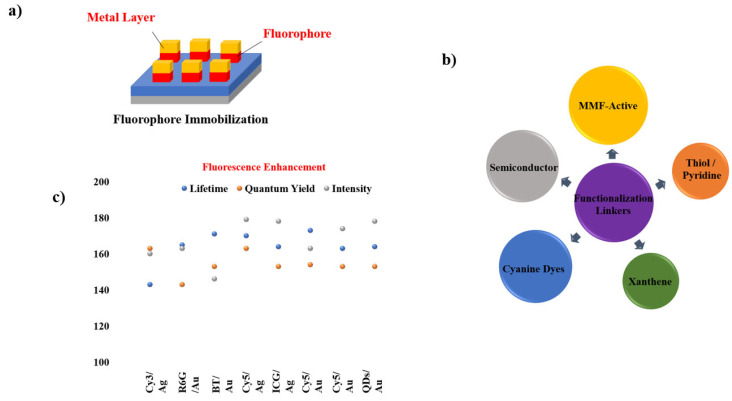
Summary of materials fabricated as Electron beam lithography for MMF studies. (**a**) Electron beam lithography method MMF structure consisting of substrate, metal layer (Ag [63,66,67,69], Au [64,65,68,69,70]), and fluorophore (Cy3/Cy5 [63,66,68,69], R6G/4-Mpy [64], BT [65], Fluorescein [66], ICG [67], QDs [70]). (**b**) Functional linkers (MMF-Active [49,145,146,147,148,150], Thiol/Pyridine [146,147], Xanthene [146,147], Cyanine Dyes [49,145,146,150], Semiconductor [151]) chemistries employed for fluorophore immobilization on metal surfaces. (**c**) Comparison of fluorescence intensity, quantum yield, and lifetime for different metal systems.

This capability makes EBL particularly valuable for MMF research, where precise control over nanoparticle geometry, spacing, and arrangement is essential for tuning plasmonic interactions and achieving reproducible fluorescence modulation [142,143,144]. Representative MMF studies employing EBL-fabricated architectures, along with their key optical outcomes, are summarized in Figure 23, with the details presented concisely in Table 10.

**Table 10:** Summary of the MMF materials and the fabrication processes employing electron beam lithography techniques, mentioning the associated experiments’ difficulties and the corresponding references.

S.No	Challenges	Ref.
1	Accurate plasmon resonance modelling for complex substrates and geometries. The ability to observe nanoscale effects was limited by the resolution of fluorescence imaging. EBL is resource-intensive	[145]
2	Lift-off is problematic with closed or complicated shapes Thicker resists were needed for thicker Au layers which decreased lithographic resolution. To ensure purity, cleaning post-etching residues needed multi-step procedures.	[146]
3	The pixel-by-pixel exposure of EBL limits throughput and time consumption Lift-off and etching at the nanoscale can be challenging and may affect structural integrity.	[147]
4	Small features (less than 70 nm) displayed slight fabrication variations. Plasmon coupling resulted in very short interparticle spacings (<210 nm), producing broader spectra and lesser enhancements.	[148]
5	Stability and variability problems, though mitigated, still existed Enhancement factor requires careful guidance; it is sensitive to several conditions.	[149]
6	Interplay between multiple factors like geometric configuration, isolation distance, and dielectric effects make optimization challenging.	[49]
7	At short distances, quenching occurs Neutral density filters and dynamic range modification are necessary to reduce high-intensity signals, complicating signal collection further	[150]
8	By precisely prepping surfaces and applying a chromium layer to improve Au-silicon bonding, adhesion problems were resolved. Non-specific adsorption, which disrupts self-assembly by binding unwanted species, was observed.	[151]

### 6.11. Summary of the Features of the Synthesis Protocols

MMF has been realized through a diverse range of synthesis strategies, each employing different materials, design philosophies, optimization approaches, and structural configurations. These methods vary widely in their advantages and limitations, and together they form a comprehensive toolbox for tailoring fluorescence lifetime, quantum yield, and emission intensity through plasmonic interactions.

Sandwich coatings represent the most fundamental MMF architecture, in which fluorophores (e.g., Tinopal, FITC, DCM, Rhodamine B/800) are positioned between plasmonic films such as silver island films (SiFs), Cu, Fe, Au, or indium. This simple and easily constructed configuration offers tunable optical properties and can produce substantial enhancements—often up to 100%. However, sandwich coatings suffer from low surface stability, poor reproducibility, and challenges in maintaining a consistent metal–fluorophore separation. Depending on film thickness, fluorophore–metal distance, and resonance overlap, lifetimes typically decrease by 10–48%, while intensities may increase by up to 100%.

Spin coating is well suited for large-area applications, enabling rapid, scalable, and relatively uniform film formation at low cost. It has been widely used with fluorophores such as Cy5-DNA and CdTe quantum dots in combination with Ag or Au nanoparticles. Key tunable parameters include spin speed and solvent evaporation rate, with achievable MMF enhancements ranging from 16 to 85 include uneven film thickness, nanoparticle aggregation, and quenching effects that reduce reproducibility.

Langmuir–Blodgett (LB) films, an extension of the Langmuir technique, provide exceptional control over molecular spacing and film thickness. They are particularly valuable for distance-dependent MEF studies, pairing metals such as Ag and Au with dyes including Ir (III) complexes and Alexa-647. Despite their precision, LB films face challenges such as low reproducibility, spectral overlap issues, interference effects, and difficulty in producing homogeneous large-area films. Typical outcomes include lifetime reductions of 30–62% and intensity increases of 20–70% when using PMMA or silica spacer layers.

Dip coating offers an inexpensive and scalable route for fabricating MMF structures using Cu, Au, or Ag nanoparticles with fluorophores such as Cy5, Oxazine 725, and various near-infrared dyes. It is particularly favourable for NIR amplification and for coating large surfaces. Enhancements up to 90% have been reported. However, oxidation of reactive metals (especially Cu), low film uniformity, and limited control over nanoparticle morphology can lead to unstable fluorescence signals.

Self-assembled monolayers (SAMs) based on alkanethiols on Au or Ag enable precise fluorophore placement and spacing, making them highly suitable for NIR/NIR-II imaging and molecular detection. SAMs offer excellent repeatability, ordered fluorophore arrangement, and can achieve dramatic enhancements—up to 400× intensity and 97% quantum yield. Their limitations include photobleaching, quenching at very small separations, and irregular morphologies when applied to complex nanostructures such as nanostars or nanodots.

Electroless deposition (ELD) allows current-free metal coating (Ag, Au, Ni) and supports fluorophores such as ICG and quantum dots. ELD can produce enhancements up to 500× and lifetime shortening up to 65%, driven by optimized surface morphology and uniform metal coverage. Strengths include high fluorescence gain, homogeneity, and compatibility with complex geometries. Weaknesses include nonspecific adsorption, over-deposition, and quenching from uncontrolled nanoparticle aggregation.

Sputter coating, a vacuum-based thin-film deposition method, enables precise control over metal thickness and structure using metals such as Cu and Sn with fluorophores like Rhodamine-6G and Cy5. Enhancements up to 40× and lifetime reductions of up to 81% have been reported. The method offers excellent stability, strong adhesion, and tunable thickness, though it may introduce surface roughness and nanostructure reshaping at very small fluorophore–metal distances.

Photolithography enables large-area fabrication of ordered Au/Ag plasmonic arrays. Fluorophores such as Rhodamine B and coumarin exhibit directional emission, 15–30% lifetime reductions, and 30–90% intensity increases. Quantum yield improvements arise from optimized resonance overlap. However, below ~100 nm feature sizes, diffusion-limited processes constrain resolution.

Layer-by-layer (LbL) assembly provides molecular-level precision in controlling fluorophore–metal spacing using polyelectrolytes, proteins, and nanoparticle layers. It offers high reproducibility and tunability in thickness and porosity. Typical outcomes include lifetime control of 10–35%, intensity increases of 40–100%, and adjustable quantum yield. Limitations include dye aggregation, complex preparation steps, and poor long-term stability.

Electron beam lithography (EBL) produces nanometer-resolution Au/Ag nanostructures with fluorophores such as Rhodamine 6G and FITC. Enhancements of 50–150% and lifetime reductions of 20–40% are common. While EBL offers unmatched spatial precision and reproducibility, its high cost and limited scalability restrict widespread use.

Across these lithographic methods—including EBL and nanoimprint lithography—extremely accurate plasmonic nanostructures (e.g., nanocages, nanorods, bowties) have been fabricated in combination with quantum dots and other fluorophores. These approaches are ideal for high-end biosensing and diagnostics due to their exceptional reproducibility and enhancement factors reaching up to 1000×. Optimization typically involves tuning spacer thickness (3–9 nm), nanostructure geometry, and grating depth, often guided by electromagnetic simulations such as FDTD and RCWA. Nonetheless, quenching remains unavoidable at metal–fluorophore distances below ~50 nm, and the methods remain costly, time-consuming, and throughput-limited.

**Figure 24:**
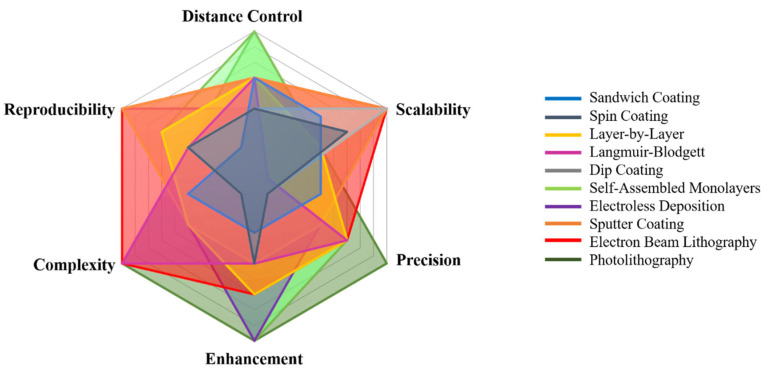
Different aspects of MMF coating methods evaluated based on the parameters including distance control, reproducibility, scalability, precision, enhancement and complexity for MMF applications.

**Figure 25:**
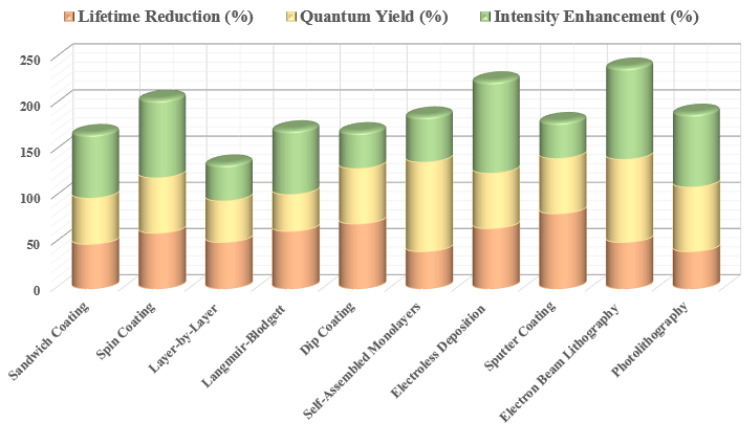
Comparison of different coating methods and fabrication techniques based on the fluorescence attributions including lifetime, intensity, quantum yield enhancing their influence on MMF performance.

**Figure 26:**
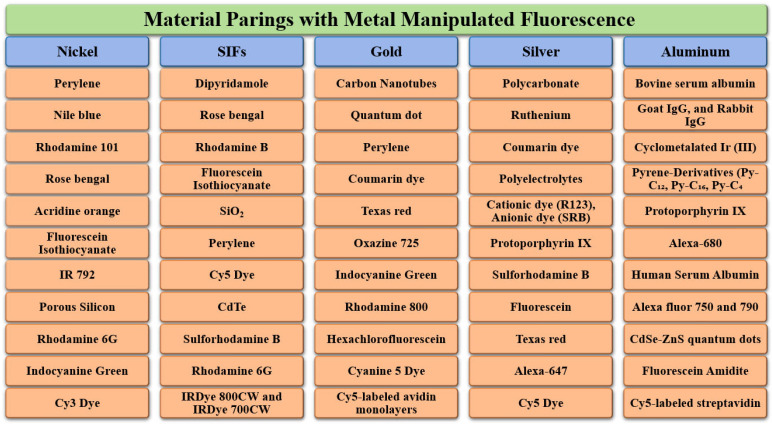
Material pairings with MMF that shows different combinations of fluorophore, dyes and the plasmonic nanomaterial.

To consolidate these findings, Figure 24 and Figure 25 provide two visual summaries. Figure 16 offers a broad overview of the synthesis processes, highlighting practical considerations such as scalability and structural control in addition to the core fluorescence metrics. Figure 17 quantifies changes in fluorescence lifetime, quantum yield, and intensity across the various methods, serving as a concise reference for researchers selecting or comparing MMF strategies. Finally, Figure 26 presents an essential summary of the reported combinations of plasmonic materials and fluorophores, offering a practical guide for designing future MMF systems.

In summary, each MMF coating strategy offers distinct advantages that make it valuable for specific applications. Spin and dip coating provide scalable, accessible platforms for large-area fabrication; SAMs and EBL deliver exceptional enhancement factors and nanoscale precision; and LbL, LB, and ELD techniques enable finely tunable, distance-sensitive architectures. Despite these strengths, the development of next-generation sensing and imaging technologies—the primary beneficiaries of MMF—will continue to depend on carefully balancing enhancement efficiency, structural control, cost, and reproducibility. The optimal choice of synthesis method will therefore remain context-dependent, guided by the practical trade-offs inherent to each approach.

## 7. Conclusions and Outlook

Metal-Manipulated Fluorescence (MMF) has emerged as a versatile and powerful platform for tailoring emission characteristics through plasmon–fluorophore interactions. Decades of systematic research have demonstrated that parameters such as fluorophore–metal spacing, nanostructure geometry, dielectric environment, and fabrication protocol critically determine whether fluorescence is enhanced or quenched. The ability to modulate intensity, lifetime, quantum yield, and photostability makes MMF highly attractive for both fundamental photophysics and a wide range of applied technologies.

This review has highlighted how diverse fabrication strategies—including sandwich coatings, spin coating, SAMs, LB films, dip coating, sputter coating, EBL, ELD, LbL assembly, and lithographic structuring—each offer distinct advantages and limitations in producing plasmonically active substrates. Collectively, these approaches have advanced MMF not only as a tool for probing nanoscale light–matter interactions, but also as a generalizable strategy for engineering sophisticated optical platforms.

Despite this progress, several challenges must be addressed before MMF can be fully translated into robust, deployable technologies. Reproducibility remains a major concern: small variations in nanoparticle morphology, fluorophore orientation, environmental conditions, or material stability can lead to large discrepancies in enhancement factors. Scalability is another limitation, as many of the highest-performing nanostructures rely on lithography or other advanced deposition techniques that are not easily adapted to low-cost, large-area manufacturing. Stability is equally critical—MMF systems must resist photobleaching and remain functional in complex or harsh environments, particularly for biological or environmental applications.

Different fabrication methods offer complementary strengths. Sandwich and spin coating provide reproducibility, accessibility, and multiplexing capability through controllable film thickness and morphology. LB assembly offers precise control of fluorophore–metal separation, enabling mechanistic and distance-resolved fluorescence studies, though it is sensitive to processing conditions. Dip coating and SAMs are inexpensive and scalable, but often limited by uniformity and long-term stability due to their reliance on molecular-scale interactions. ELD enables large-area metallic coatings without complex equipment, but requires careful control to prevent aggregation. Sputter coating yields uniform thin films but may induce quenching at very small separations. Photolithography and EBL provide unmatched precision and are indispensable for fundamental studies and high-performance platforms, yet remain costly and difficult to scale.

Balancing performance, reproducibility, and scalability, we conclude that spin coating, sandwich coating, and LbL assembly currently represent the most promising routes for cost-effective MMF platforms. Spin coating offers simplicity, controllability, and scalability for device manufacturing, while sandwich coatings allow straightforward tuning of thickness to balance enhancement and quenching. LbL assembly provides molecular-level precision in fluorophore–metal spacing, making it ideal for controlled MMF studies. Meanwhile, photolithography and EBL remain essential for defining the structural limits of nanophotonics, guiding future innovations that may eventually translate these capabilities into scalable formats.

The potential impact of MMF spans multiple fields. In environmental sensing, plasmon–fluorophore systems can enable ultra-sensitive detection of trace contaminants and hazardous gases, addressing urgent challenges in air and water quality monitoring. In biomedicine, MMF-based probes promise brighter imaging, improved diagnostic sensitivity, and enhanced therapeutic monitoring through reduced photobleaching and higher signal-to-noise ratios. In photonics and energy, MMF architectures can contribute to efficient solar energy harvesting, tunable light sources, and integrated photonic circuitry. Realizing these advances will require a fusion of precise fabrication control and predictive computational design, supported by tools such as FDTD, DDA, and DFT for structural and optical optimization.

In conclusion, MMF stands as both a powerful platform for exploring nanoscale light–matter interactions and a promising pathway toward next-generation sensing and optoelectronic technologies. Among the many fabrication techniques available, spin coating, sandwich coating, and LBL assembly currently offer the best balance between enhancement performance and practical manufacturability. As fabrication methods mature, computational design becomes more sophisticated, and interdisciplinary integration accelerates, MMF is poised to transition from laboratory demonstrations to real-world applications. With its unique ability to modulate fluorescence from quenching to strong enhancement within a single platform, MMF is set to play a central role in the development of future sensors, diagnostic tools, and photonic systems.

The authors note that apart from the specific summaries given so far, multiple avenues that are both interesting and can be quite revealing are yet to be fully explored. The first and foremost will be in coming up with frameworks of fabrication that are universal. To be more specific, this means making available standard methods of fabrication that are repeatable across labs globally, similar to the commercial processes of manufacturing. This is an often unsought direction, probably because of the fact that the drive towards benchmarking often supersedes that of fabrication process optimization. We stress that the latter is equally important, as often, if global standards of material fabrication are available, benchmarking can proceed even more rapidly because of global and not local investigations. Secondly, the drive towards understanding the mechanistic aspects, through time-resolved studies, might be strived to be attempted to at least reasonable degrees of shape and form by every study, in order to continually refine and discover Plasmon manipulative mechanisms of fluorescence. Thirdly, the field of metallic alloys for fluorescence tuning remains relatively new. This is proposed by the authors as a promising direction in order to circumvent/synergise the material properties of multiple metals, for example. Fourthly, we propose that a plasmon-mediated photon upconversion might be explored for augmenting fluorescence. Specifically, the documented ability of plasmons to upconvert incident photons in the near-infrared for example might be researched on to study the resulting improvements to fluorescence emissions when the upconverted photon is channelled into the fluorophore. Finally, hybrid methods of fabrication, with template-based synthesis being cost-prohibitive and solution-based ones having low fidelity, can offer merits of both. For example, flow-assisted anchoring of solution-synthesized plasmonic particles on template-based scaffolds can be an exploratory way forward. The thus stated suggestions might pave new paths for this interesting field of metal-manipulated fluorescence.

## Data Availability

No new data were created or analyzed in this study.
